# Contribution of single amino acid and codon substitutions to the production and secretion of a lipase by *Bacillus subtilis*

**DOI:** 10.1186/s12934-017-0772-z

**Published:** 2017-09-25

**Authors:** Pia Skoczinski, Kristina Volkenborn, Alexander Fulton, Anuseema Bhadauriya, Christina Nutschel, Holger Gohlke, Andreas Knapp, Karl-Erich Jaeger

**Affiliations:** 1 Institute of Molecular Enzyme Technology, Heinrich-Heine-University Düsseldorf, 40225 Düsseldorf, Germany; 2 Institute for Pharmaceutical and Medicinal Chemistry, Heinrich-Heine-University Düsseldorf, 40225 Düsseldorf, Germany; 3 John von Neumann Institute for Computing (NIC), Jülich Supercomputing Centre (JSC) & Institute for Complex Systems - Structural Biochemistry (ICS6), Forschungszentrum Jülich GmbH, 52425 Jülich, Germany; 4 Institute of Bio- and Geosciences IBG-1: Biotechnology, Forschungszentrum Jülich GmbH, 52425 Jülich, Germany; 5 Present Address: Macromolecular Chemistry and New Polymeric Materials, Zernike Institute of Advanced Materials, University of Groningen, Nijenborgh 4, 9747AG Groningen, The Netherlands; 6 Present Address: Novozymes A/S, Krogshoejvej 36, 2880 Bagsvaerd, Denmark

**Keywords:** *Bacillus subtilis*, Lipase, Protein production, Secretion, Optimization

## Abstract

**Background:**

*Bacillus subtilis* produces and secretes proteins in amounts of up to 20 g/l under optimal conditions. However, protein production can be challenging if transcription and cotranslational secretion are negatively affected, or the target protein is degraded by extracellular proteases. This study aims at elucidating the influence of a target protein on its own production by a systematic mutational analysis of the homologous *B. subtilis* model protein lipase A (LipA). We have covered the full natural diversity of single amino acid substitutions at 155 positions of LipA by site saturation mutagenesis excluding only highly conserved residues and qualitatively and quantitatively screened about 30,000 clones for extracellular LipA production. Identified variants with beneficial effects on production were sequenced and analyzed regarding *B. subtilis* growth behavior, extracellular lipase activity and amount as well as changes in lipase transcript levels.

**Results:**

In total, 26 LipA variants were identified showing an up to twofold increase in either amount or activity of extracellular lipase. These variants harbor single amino acid or codon substitutions that did not substantially affect *B. subtilis* growth. Subsequent exemplary combination of beneficial single amino acid substitutions revealed an additive effect solely at the level of extracellular lipase amount; however, lipase amount and activity could not be increased simultaneously.

**Conclusions:**

Single amino acid and codon substitutions can affect LipA secretion and production by *B. subtilis*. Several codon-related effects were observed that either enhance *lipA* transcription or promote a more efficient folding of LipA. Single amino acid substitutions could improve LipA production by increasing its secretion or stability in the culture supernatant. Our findings indicate that optimization of the expression system is not sufficient for efficient protein production in *B. subtilis*. The sequence of the target protein should also be considered as an optimization target for successful protein production. Our results further suggest that variants with improved properties might be identified much faster and easier if mutagenesis is prioritized towards elements that contribute to enzymatic activity or structural integrity.

**Electronic supplementary material:**

The online version of this article (doi:10.1186/s12934-017-0772-z) contains supplementary material, which is available to authorized users.

## Background

The Gram-positive soil bacterium *Bacillus subtilis* secretes up to 20 g/l of produced proteins directly into the culture supernatant [1, 2]. Therefore, it has become more and more important in industrial applications for the production of homologous and heterologous proteins in large-scale fermentation processes [1]. Due to this fact, *B. subtilis* has been intensively studied and optimized as a protein production host in the last decades, establishing it as a ‘microbial cell factory’ [3, 4].

Optimization strategies have targeted several bottlenecks for heterologous protein production in *B. subtilis*. Examples include optimization of transcription efficiency by using strong promoters such as the constitutive promoter P_*aprE*_ or an arabinose-inducible promoter [3]. Fine-tuning of translation [5] can be achieved by either using optimized ribosome binding sites to improve ribosome binding of the mRNA [3] or by introducing translational pauses using ‘slow-translating’ codons, as previously shown for heterologous protein production in *E. coli* [5, 6].

The majority of secretory proteins in *B. subtilis* are targeted to the Sec translocon and translocated via the cotranslational Sec-SRP pathway [7–10]. To optimize the protein secretion step as a prospective bottleneck, several studies assayed for the optimal signal peptide necessary for secretion. Screening a set of 173 Sec-specific signal peptides of *B. subtilis* [11] or the additional screening of heterologous signal peptides from *B. licheniformis* [12] successfully identified signal peptides for improved secretion of the *Fusarium solani pisii* cutinase [11] and the *B. amyloliquefaciens* subtilisin BPN’ [13] in *B. subtilis*. Maturation and folding of secreted proteins are increased by the overexpression of regulatory factors, e.g. the lipoprotein PrsA, which resulted in increased secretion rates of α-amylase of *B. stearothermophilus* by *B. subtilis* [14]. Furthermore, strains lacking the majority of the major extracellular proteases have been constructed, e.g. the *B. subtilis* strain WB800 lacking all eight extracellular proteases (AprE, NprE, NprB, Vpr, Bpr, Mpr, Epr, WprA), resulting in strongly decreased degradation of extracellular target proteins [2]. A few studies with Gram-negative bacteria indicated that the target protein itself can also influence its production and secretion, e.g. by interactions with the translocation machinery [15, 16]. However, no systematic study has yet been reported on the role of each amino acid of a secreted protein for its production and secretion. Here, we have systematically analyzed single amino acids and their respective codons of *B. subtilis* lipase A (LipA) to understand beneficial and detrimental effects of amino acid and codon substitutions on LipA production and secretion.

The extracellular lipase LipA is one of the smallest known lipases showing a minimal α/β-hydrolase fold consisting of six β-sheets and six α-helices [17]. Compared to the classical α/β-hydrolase fold, two β-sheets are missing, the αD-helix is substituted by a small 3_10_-helix, and the αE-helix contains only four amino acids [17]. LipA features a surface-exposed active site consisting of amino acids S77, D133 and H156, which is accessible for the substrate without conformational change; the oxyanion hole is formed by I12 and M78 [17, 18]. LipA is secreted cotranslationally via the Sec-SRP pathway. The N-terminal signal peptide is cleaved off by a signal peptidase resulting in the mature enzyme with 181 amino acids and a molecular weight of 19.34 kDa [8, 19].

LipA was subjected to a nearly complete site saturation mutagenesis targeting 155 of 181 residues with a conservation < 95% within the *Firmicutes* phylum. The resulting library was screened for extracellular lipase production both qualitatively and quantitatively. Our results indicate that both single amino acid and codon substitutions significantly affect production and secretion of the target protein and suggest that optimization studies should aim primarily at structural elements that contribute to enzymatic activity or structural integrity.

## Methods

### Bacterial strains and plasmids

Bacterial strains and plasmids used in this study are listed in Table 1. *E. coli* DH5α was used for cloning and plasmid amplification. *B. subtilis* TEB1030 was used as the secretory expression host.

**Table 1:** Bacterial strains and plasmids

Bacterial strains and plasmids	Genotype	References
Bacterial strains		
*E. coli* DH5α	*supE44 ∆(lacZYA*-*argF) U196 (phi80∆lacZM15) hsdR17 recA1 endA1 gyrA96 thi*-*1 relA1*	[20]
*B. subtilis* TEB1030	*trpC2 his nprE aprE bpf ispI lipA lipB*	[19]
Plasmids		
pBSMul1	*E. coli*–*B. subtilis* shuttle vector, ribosome binding site, P_*HpaII*_, secretion (*sslipA*) and purification (C-terminal 6x-His-tag); ColE1 *repB Km* ^*r*^ *Amp* ^*r*^	[21]
pET22lipA	pET22b (Novagen, USA) containing a 557 bp *Eco*RV/*Sac*I fragment of *B. subtilis lipA* gene fused to *pelB* signal peptide sequence, P_T7*lac*_	[18]
pBSlipA	pBSMul1 containing a 568 bp *Eco*RI/*Hin*dIII fragment of *B. subtilis lipA* gene; additionally deleted *Eco*RI restriction site	This study

### Growth of *B. subtilis*

*Escherichia coli* and *B. subtilis* were grown in LB medium (10 g/l tryptone, 10 g/l NaCl, 5 g/l yeast extract) with 100 µg/ml ampicillin or 50 µg/ml kanamycin, respectively, at 37 °C. Culture volumes, agitation speed and preparation of supernatants at different cultivation conditions are described below.

### 96-well microtiter plate cultivation

For the two-step screening procedure, *B. subtilis* was pre-cultivated in 150 µl LB medium in 96-well microtiter plates (Greiner Bio-one, Germany) at 37 °C, 900 rpm for 6 h (TiMix 5, Edmund Bühler GmbH, Germany). These pre-cultures were used to inoculate expression cultures in 150 µl fresh LB medium in 96-well microtiter plates (Greiner Bio-one, Germany) to an O.D._580nm_ of 0.05 with a TECAN^®^ robotic system freedom evo (Tecan Group Ltd., Germany). Expression cultures were cultivated at 25 °C, 900 rpm for 16 h (TiMix 5, Edmund Bühler GmbH, Germany). The cells were harvested by centrifugation (4 °C, 5000 × *g*, 30 min) and the culture supernatant was immediately used for analysis.

### Microfermentation in 48-well FlowerPlate^®^ and online biomass measurement

*Bacillus subtilis* clones were pre-cultivated in 1100 µl LB medium in 48-well Flowerplates (FlowerPlate^®^ 48 well MTP without optodes, m2p-labs, Germany) at 37 °C, 1100 rpm for 16 h (TiMix 5, Edmund Bühler GmbH, Germany). Expression cultures were inoculated to an O.D._580nm_ of 0.05 in 1100 µl LB medium in 48-well Flowerplates and cultivated at 37 °C, 1100 rpm for 6 h. For cell harvest, 50 µl of each culture were transferred into a 96-well microtiter plate (Greiner Bio-one, Germany) and centrifuged as described above.

### Transformation of *E. coli* and *B. subtilis*

Electrocompetent *E. coli* DH5α cells were prepared as previously described [22]. *E. coli* DH5α was transformed by electroporation in a MicroPulser (BioRad, Germany). *B. subtilis* TEB1030 cells were transformed by protoplast formation as previously described [23].

### Construction of the *lipA* expression vector pBSlipA, site saturation mutagenesis and library construction

The *lipA* gene (KEGG Accession Number BSU02700) without its native signal sequence was amplified from the *E. coli* expression vector pET22lipA [18] using the oligonucleotides *Eco*RI_fw (5′ cgcggaattcgctgaacac 3′) and *Hin*dIII_rev (5′ agtgcggccgcaagcttgtcgacgtaatgttcattaattcgtatt 3′). The resulting 568 bp *Eco*RI/*Hin*dIII fragment was cloned in frame with the native *lipA* signal sequence (*sslipA*) under the control of the strong constitutive promoter P_*Hpa*II_ in the *E. coli*–*B.* *subtilis* shuttle vector pBSMul1 [21] previously used for analysis of secretory protein production [11, 13]. The additional six base pair linker of the *Eco*RI restriction site between the *sslipA* and the *lipA* gene was subsequently deleted by QuikChange^®^ PCR [24] using the primer pair ∆*Eco*RI_fw (5′ agcaaaagccgctgaacacaatc 3′) and ∆*Eco*RI_rev (5′ gattgtgttcagcggcttttgct 3′). The generated expression vector pBSlipA harbors a native full-length *lipA* gene and was used for *lipA* expression and mutagenesis.

Oligonucleotide design and site saturation PCR were performed as previously described [25]. In short, the vector was amplified with degenerated ‘NNS’ oligonucleotides (Additional file 1: Table S1) by QuikChange^®^ PCR [24]. The remaining template vector DNA in the PCR product was hydrolyzed using *Dpn*I, and the site saturation PCR product was desalted and concentrated by PCR Purification Kit (Analytik Jena, Germany). First, *E. coli* DH5α was transformed by electroporation, and the mutagenesis vectors were isolated from 2000 to 4000 *E. coli* clones. Subsequently, the secretory protein production strain *B. subtilis* TEB1030 was transformed with 20 ng of vector DNA by protoplast formation.

To achieve a library coverage of about 99.9%, 192 clones are necessary for each position, i.e. six-times the number of codons (32 via ‘NNS’) as described in [26]. Thus, a library for the site saturation mutagenesis of a certain position was distributed to two 96-well plates. However, we reduced the clone number to 184 *B. subtilis* TEB1030 transformants allowing to add 8 wild-types and negative controls. Taking into account that mutagenesis could also re-introduce the wild-type codon, a set of 184 transformants per residue leads to a full coverage probability of 93.87% calculated with TopLib (http://stat.haifa.ac.il/~yuval/toplib/) [27] and a supposed mutagenesis yield of 90%.

Double mutants were constructed by site directed PCR following the procedure described above for site saturation PCR. Oligonucleotides for site directed mutagenesis are listed in Additional file 1: Table S2.

### Lipase activity assay with *B. subtilis* culture supernatant

Extracellular lipase activity was determined in 96-well microtiter plates (Greiner Bio-one, Germany). The *B. subtilis* culture supernatant obtained by centrifugation was mixed with *para*-nitrophenyl palmitate (*p*NPP) substrate solution as previously described [11], and hydrolysis of *p*NPP was measured spectrophotometrically (λ_abs_ = 410 nm) at 37 °C for 15 min using the plate reader SpectraMax 250 (Molecular Devices, Germany). Lipolytic volume activity was calculated using a molar extinction coefficient of 15,000 M^−1^ cm^−1^. Specific lipase activity (U/mg) was calculated by the volume activity (U/ml) per protein amount (mg/ml). The LipA protein amount was quantified as described in the next paragraph. Unless stated otherwise, a two-tailed t-test was performed with a significance level of *p* < 0.05 to determine significant activity changes.

### Enzyme-linked immunosorbent assay with *B. subtilis* culture supernatant

For quantitative detection of extracellular LipA protein, an enzyme-linked immunosorbent assay (ELISA) using a specific polyclonal LipA antibody (Eurogentec, Germany) was performed. 15.6 μl twofold prediluted *B. subtilis* culture supernatant obtained by centrifugation was diluted in 100 μl bicarbonate buffer (100 mM; pH 9.6) and transferred into Polysorp^®^ 96-well microtiter plates (Nunc-Immuno™ MicroWell™ 96-Well Plate) using the TECAN^®^ robot system. After coating of proteins onto the plastic surface at 4 °C, 100 rpm for 22 h and three times washing with PBS (10 mM phosphate-buffered saline; pH 7.4), blocking with 1% (w/v) bovine serum albumin (BSA) diluted in PBS was performed at 22 °C, 150 rpm for 2.5 h. Plates were washed two-times with PBS and polyclonal rabbit anti-LipA antibody diluted 1:5000 in PBS was added and incubated at 22 °C, 150 rpm for 2 h, followed by four times washing with PBS. After another 3 h incubation with the goat anti-rabbit horseradish peroxidase antibody (diluted 1:5000 in PBS; BioRad, Germany), Polysorp^®^ 96-well microtiter plates (Nunc-Immuno™ MicroWell™ 96-Well Plate) were finally washed four times with PBS.

LipA was quantified by determination of horseradish peroxidase activity measured using the 1-step TMB ELISA substrate (3,3′,5,5′-tetramentylbenzidine; Thermo Fisher Scientific, Germany) at 25 °C for 15 min in the SpectraMax 250-plate reader (Molecular Devices, Germany). The amount of extracellular LipA was calculated using a standard curve determined with purified LipA. A two-tailed t-test was performed with a significance level of *p* < 0.05 to determine significant changes in LipA protein amount.

### Real-time quantitative PCR for determination of *lipA* transcripts

Cell cultures were harvested after 6 h of growth, and RNA was prepared using the NucleoSpin^®^ RNA Kit (Macherey–Nagel, Germany). cDNA synthesis of 1 µg RNA was performed with the Maxima First Strand cDNA Synthesis Kit for RT-qPCR Kit (Thermo Fisher Scientific, Germany). 50 ng cDNA and 50 ng of RNA (NoRT controls) were applied for RT-qPCR using the Maxima SYBR/ROX qPCR Master Mix (Thermo Fisher Scientific, Germany) and the primer pairs *lipA*_fw: *5*′gcttccgggaacagatccaa 3′ and *lipA*_rev: 5′ acagaaggccgatgtgtcca 3′. The *sigA* gene was used as a reference and amplified using the primers *sigA*_fw: 5′ atcgcctgtctgatccacca 3′ and *sigA*_rev: 5′ ggtatgtcggacgcggtatg 3′. Gene expression analysis was performed with the REST 2009 software (Qiagen, Germany) using the 2^−ΔΔCT^ method with an assumed PCR efficiency of 100% [28, 29]. Here, expression of *lipA* in three biological replicates (each analyzed three-times by RT-qPCR) is first normalized to the expression level of the reference gene *sigA* in the same culture, which encodes for the major sigma factor in *B. subtilis* and is equally expressed in all cells with less than 5% deviation in all analyzed samples. In a second step, the resulting value is compared to the corresponding value derived from a control culture, here *B. subtilis* expressing the wild-type *lipA* gene, resulting in an x-fold change in expression level.

To obtain information about the reliability and reproducibility of the RT-qPCR data, the relative change of normalized *lipA* transcript amount among all wt*lipA* expressions was determined using the REST 2009 software (Qiagen, Germany). 33 replicates were analyzed twice and revealed a standard error for the wt*lipA* transcript amount of 0.6 or 1.2 (lower and upper standard error, respectively). Therefore, only changes of transcript amounts lower than 0.4 or higher than 2.2 with a *p* value < 0.05 (calculated by REST 2009) were defined as significantly changed.

### Sequence analysis

Protein sequences were obtained from the Pfam database of protein families [30] to determine the degree of amino acid conservation with respect to *B. subtilis* LipA. 64 lipase (Class 2) sequences out of 41 species from the *Firmicutes* phylum were aligned using Clustal Omega [31]. The number of amino acids in this alignment identical to the amino acid in the *B. subtilis* LipA sequence was counted for each position. This position-dependent conservation of each *B. subtilis* LipA amino acid within the *Firmicutes* phylum in percent is shown in Additional file 1: Table S3. The hydropathy index of Kyte and Doolittle was used as hydrophobicity scale [32] and changes > 1 were assumed to be significant.

### Constraint network analysis

The X-ray crystal structure (PDB ID: 1ISP) with the highest resolution (1.3 Å) of *B. subtilis* LipA was used as the wtLipA structure, as well as a template to generate structures for LipA variants. All buffer ions and crystallization solvents were removed from the crystal structure. The models of the single variant structures were generated by the SCWRL4 program [33]. With the help of a rotamer library, SCWRL4 constructs variant models by predicting backbone-dependent side-chain conformations, while coordinates of backbone atoms stay unchanged. For enabling a local structural relaxation around the mutated residue, conformations of side chains of all residues within 8 Å of the mutated residue were re-predicted. Hydrogen atoms were added, and side chains of Asn, Gln and His were flipped by the REDUCE program [34] for all variant structures. All structures were minimized by 100 steps of steepest descent followed by 5000 steps of conjugate gradient minimization or until the root mean-square gradient of the energy was < 1.0 * 10^−4^ kcal mol^−1^ Å^−1^. The energy minimization was carried out with Amber14 using the ff99SB force field [35] and the GB^OBC^ Generalized Born model [36].

Thermal unfolding simulations by constraint network analysis (CNA) were performed as described previously [37–39]. In order to improve the robustness of CNA but without comprising CNA’s high computational efficiency, CNA was carried out on an ensemble of network topologies generated from a single input structure by using fuzzy non-covalent constraints [40]. Here, the number and distribution of non-covalent constraints are modulated by random components within the ranges described in the Additional file 2: Methods, thus simulating thermal fluctuations of a biomacromolecule without actually moving atoms. An ensemble of 1000 network configurations was generated for wtLipA and all LipA variants. For the thermal unfolding simulations, the hydrogen bond energy cutoff *E*_cut_ was varied between −0.1 to −6.0 kcal mol^−1^ with a step size of 0.1 kcal mol^−1^, equivalent to increasing the temperature from 302 to 380 K in steps of 2 K [41]. The number of hydrophobic constraints was kept constant during the thermal unfolding simulations.

A neighbor stability map [42] averaged over all 1000 conformations was computed from the thermal unfolding trajectories, and its median ($\widetilde{rc}_{{{\text{ij, }}neighbor}}$) was used to compare the thermostabilities of wtLipA and LipA variants, as done previously [43]. See Additional file 2: Methods for more information.

## Results

### Construction of the *lipA* site saturation mutagenesis library

**Fig. 1:**
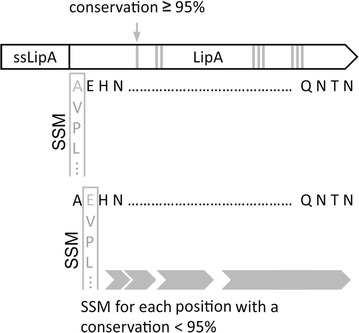
Schematic strategy to construct the *lipA* site saturation mutagenesis library. The expression vector pBSlipA, where the native *lipA* gene is under control of the constitutive P_*HpaII*_ promoter, was used for construction of the *lipA* library by site saturation mutagenesis (SSM). The gene encodes the secretion signal (ssLipA) and the mature lipase (LipA). Each codon encoding an amino acid of the mature LipA with a conservation < 95% within the *Firmicutes* was considered for SSM using QuikChange^®^ PCR with a degenerated “NNS” codon to randomly introduce every possible amino acid. Amino acids with a conservation ≥ 95% (|) were not considered for substitution and screening. In total, 155 out of 181 residues were substituted and the resulting variants subjected to screening

The expression vector pBSlipA (see “Methods” section) encoding the native LipA of *B. subtilis* was used for site saturation mutagenesis (Fig. 1). In total, 155 amino acid residues of LipA with a conservation < 95% within the *Firmicutes* phylum (Pfam database entry: PF01674) [30] were used to generate the screened 29,199 clones as described in the “Methods” section.

### Two-step screening of the *lipA* site saturation mutagenesis library

**Fig. 2:**
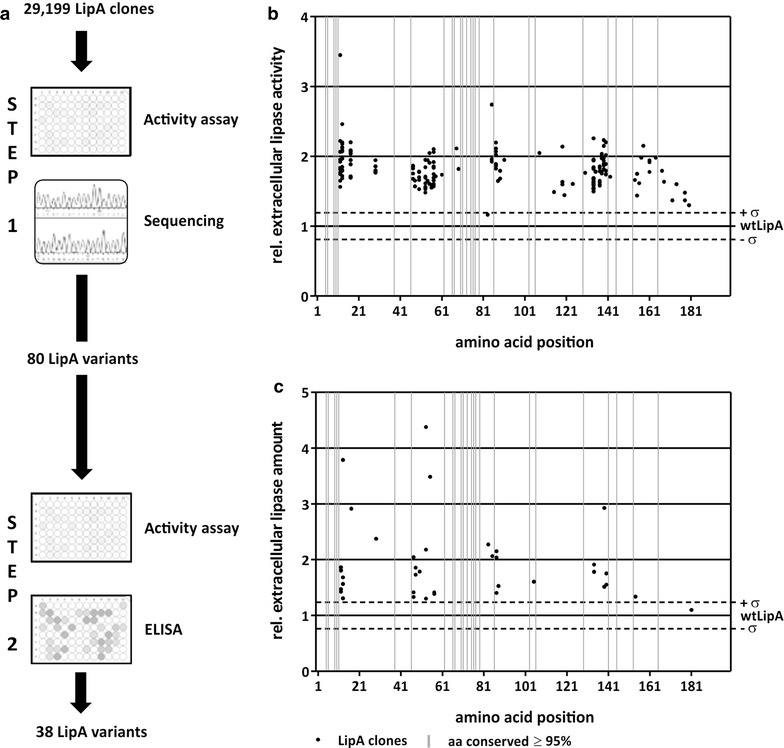
Identification of LipA variants with enhanced extracellular activity or amount of protein. **a** Schematic representation of the two-step screening procedure. In step I, 29,199 LipA clones were analyzed for increased extracellular lipase activity in the culture supernatant of *B. subtilis* TEB1030. 175 clones were sequenced and 80 LipA variants identified with increased extracellular lipase activity (see panel **b**). In a second step, culture supernatants of these variants were analyzed as nine biological replicates for increased extracellular lipase activity and protein amount. **b** 80 LipA clones with increased extracellular lipase activity. The relative extracellular lipase activity of the LipA clones is plotted against the substituted amino acid position. **c** LipA variants with increased extracellular lipase amount. The extracellular lipase amount is plotted against the substituted amino acid position. In panels **b** and **c**, each black dot represents one LipA variant, and the grey bars mark the highly conserved amino acid positions (≥ 95%). Wild-type values, which were 0.57 ± 0.12 U/ml and 3.7 ± 0.6 µg/ml, respectively, were set to 1 (wtLipA) and the grey horizontal dotted lines mark the wtLipA standard deviation (σ)

The LipA clones were cultivated in 96-well microtiter plates and analyzed with a two-step screening procedure including determination of extracellular volume activity and amount of LipA (Fig. 2a).

In the first step, extracellular lipase activity was determined with *p*NPP as the substrate. In total, 5444 clones (19%) were inactive with the majority located at amino acid positions 26, 35, 41, 49, 101, 102, 104, 156, 160 and 181. To calculate a mean wtLipA lipase activity, 384 wtLipA clones were analyzed allowing to separate clones with significantly increased or decreased extracellular lipase activity from those with wtLipA activities. The volume activity and the corresponding standard deviation (σ) of wtLipA were 0.57 ± 0.12 U/ml. Compared to this, 4230 clones (14%) showed a significant decrease in extracellular lipase activity with amino acid substitutions at positions 19, 22, and 40. Furthermore, 66% (19,350) of all 29,199 screened clones showed activities similar to that of wtLipA and were therefore discarded.

Only 175 clones (1%) produced LipA variants with volume activities that were larger than wtLipA volume activity with its standard deviation (LipA variant U/ml > wtLipA U/ml + σ). Sequencing of the respective inserts revealed 26 clones as false-positive harboring the *lipA* wild-type sequence, 65 clones as duplicates with the identical codon exchange, and four LipA clones with multiple amino acid substitutions. The resulting 80 LipA variants (Fig. 2b) showed single amino acid substitutions distributed over 38 amino acid positions and an increase in extracellular lipase activity from 1.2- to 3.4-fold in comparison to wtLipA.

Beneficial substitutions mainly accumulated between N-terminal amino acid positions 11–18, in the middle part of LipA between positions 46–59, and in the C-terminal part between positions 129–143 and 151–169, but a clear pattern regarding amino acid position or property was not obvious.

In a second step, the 80 LipA variants from step 1 (Fig. 2) exhibiting increased extracellular lipase activity were analyzed as nine biological replicates in a 96-well microtiter plate. Extracellular lipase activity was determined and extracellular lipase amount was quantified with an enzyme-linked immunosorbent assay (Fig. 2c). 31 variants turned out to be false-positives in this verification step and did not show improved activity or amount compared to wtLipA. Additional eleven variants exhibited increased lipase activity but not protein amount. The remaining 38 variants showed an increased lipase amount with increased or similar activity compared to wtLipA. These 38 variants included 34 different amino acid substitutions and four variants with a substitution caused by a synonymous codon. Their extracellular protein amount ranged from 1.3-fold (a substitution at the C-terminal amino acid position 134) to 3.8-fold (N-terminal position 13) higher than that of wtLipA, which is produced at 3.7 ± 0.6 µg/ml (Fig. 2c).

The extracellular activity and amount of the 38 LipA variants could be affected at different stages including transcription, translation, and secretion (which are coupled for LipA), and/or improved maturation, folding, and activity. We produced these LipA variants by cultivating *B. subtilis* TEB1030 in a microfermentation system linked to online biomass measurement and analyzed transcription, activity, and protein amount after 6 h when production and secretion of wtLipA had reached their optimum (Additional file 3: Figure S1). Furthermore, online biomass measurements were performed for 24 h to exclude differences in growth of variant-producing *B. subtilis* clones, which was, however, not observed (Additional file 3: Figure S2).

**Fig. 3:**
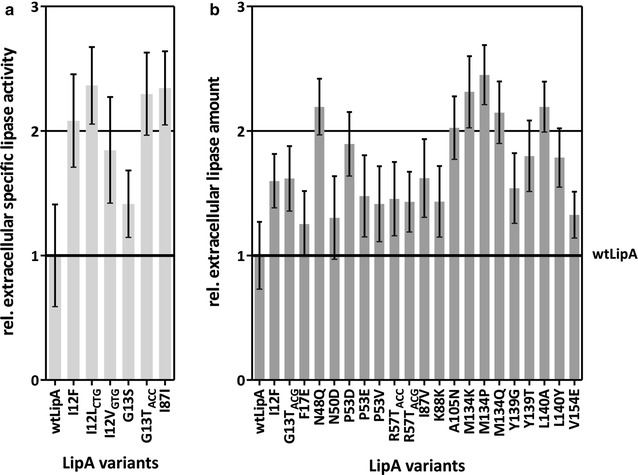
LipA variants showing increased extracellular lipase activity (**a**) or increased extracellular lipase amount (**b**). *B. subtilis* TEB1030 producing the different LipA variants were cultivated for 6 h in a 48 well Flowerplate^®^, and the relative extracellular specific lipase activity (U/mg) in the culture supernatant was calculated by normalizing the volume activity (U/ml) to the determined protein amount (mg/ml). The extracellular lipase activity was measured using *p*NPP as a substrate, and the extracellular protein amount was determined by ELISA using a specific polyclonal LipA antibody. LipA variants with significant (*p* < 0.05) increase in extracellular specific lipase activity (*n* = 9) are shown relative to wtLipA. wtLipA values were set to 1 (thick black line)

Twelve LipA variants did not show increased extracellular enzyme activity or protein amount and were therefore discarded as false positives (Additional file 1: Table S4). Six LipA variants were identified as more active with an up to 2.4-fold increase in specific lipolytic activity in comparison to wtLipA with 64 ± 13 U/mg (Fig. 3a; Additional file 1: Table S4). In total, 21 variants (including one that also showed increased activity) showed an up to 2.3-fold increase in extracellular lipase amount (Fig. 3b; Additional file 1: Table S4).

Interestingly, the increase in extracellular LipA amount and/or activity of these 26 variants is unrelated to a change in hydrophobicity of the respective amino acid: 11 LipA variants carry a substitution to a significantly less hydrophobic amino acid, amino acid substitutions of 12 LipA variants do not or only slightly change hydrophobicity, and 3 LipA variants carry substitutions to more hydrophobic amino acids (see “Methods” section).

### LipA variants with improved extracellular specific activity

Three out of the six variants with increased specific activity carry a substitution at amino acid I12 to phenylalanine, leucine, or valine, leading to a twofold increase in extracellular specific activity (Fig. 3a; Additional file 1: Table S4). LipA variants I12L_CTG_, I12V_GTG_, and G13T_ACC_ were identified as more active, whereas identical amino acid substitutions encoded by different codons either showed no effect on LipA specific activity or LipA amount (I12L_TTG_, I12V_GTC_, see Additional file 1: Table S4) or resulted in an increased LipA amount (G13T_ACG_, see Fig. 3b and Additional file 1: Table S4). Variant I87I with a silent mutation showed a twofold increase in extracellular specific activity but also a 3.6-fold significant change in *lipA* transcript level (Additional file 1: Table S4). This indicates, in all four cases, a codon- and not an amino acid-specific effect on LipA specific activity.

### LipA variants with increased extracellular lipase amount

21 LipA variants showed a 1.3- to 2.3-fold increase in extracellular LipA protein amount at predominantly similar or decreased levels of extracellular specific activity compared to wtLipA (Fig. 3b; Additional file 1: Table S4) with the exception of variant I12F, which also showed a significant twofold increase in extracellular specific lipase activity (Fig. 3a; Additional file 1: Table S4). Only the mutations G13T_ACG_ and I87I showed a significant 2.7- or 3.6-fold change in *lipA* transcript amount, respectively, while the transcript amount of all other 19 LipA variants was not significantly changed compared to wt*lipA* transcript (Additional file 1: Table S4).

We identified two LipA variants with the identical amino acid substitution R57T, which were encoded by the codons ACC and ACG (Fig. 3b; Additional file 1: Table S4). Both variants showed a similar increase in the extracellular LipA amount of ca. 1.4-fold compared to wtLipA level, indicating that this effect is caused by the introduced amino acid and not by the codon.

Seven LipA variants (N50D, P53D, P53E, P53 V, R57T_ACC_, R57T_ACG_ and M134Q) with increased extracellular LipA amount have amino acid substitutions located either in the αB-helix of LipA or carry a substitution to glutamine at position 134 (M134Q) (Fig. 3b; Additional file 1: Table S4). Since position M134 is known to contribute to thermostability [44] and the αB-helix also plays a role in tolerance towards detergents and ionic liquids [25, 45], (thermo)stability simulations were performed to probe for changes on LipA’s (thermo)stability.

### Thermal unfolding simulations of LipA variants

In order to determine to what extent an increase in LipA (thermo)stability could contribute to an increased extracellular LipA amount, the five variants N50D, P53D, P53E, P53V, and R57T with amino acid substitutions in the αB-helix and variant M134Q were subjected to thermal unfolding simulations by constraint network analysis [38]. CNA is a rigidity theory-based approach that models proteins as networks of constraints, where the constraints are defined from covalent and non-covalent (hydrogen bonds and hydrophobic interactions) bonds in the protein. Thermal unfolding of the protein is then simulated by removing hydrogen bond constraints in a step-wise manner in the order of increasing strength [41], and the influence on protein structural stability is monitored by global and local rigidity indices [42]. Here, as done previously for LipA [39, 46], the thermodynamic thermostability of LipA variants is compared to wtLipA in terms of a local index, the median of the neighbor stability map $\widetilde{rc}_{{{\text{ij, }}neighbor}}$. This $\widetilde{rc}_{{{\text{ij, }}neighbor}}$ has been shown to be related to the experimental melting temperature (*T*_m_) and to be robust if variants follow different unfolding pathways [46]. Compared to the wtLipA $\widetilde{rc}_{{{\text{ij, }}neighbor}}$ value of 316.1 K, the variants N50D, P53E, P53V, R57T and M134Q show a decrease in thermodynamic thermostability by about 1.5 K on average (Table 2).

**Table 2:** Constraint network analysis (CNA) of wtLipA and LipA variants

LipA variants	$\widetilde{rc}_{{{\text{ij, }}neighbor}}$ (K)^a^	$\Delta \widetilde{rc}_{{{\text{ij, }}neighbor}}$ (K)^b^
wtLipA	316.1	–
N50D	312.1	−4.0
P53D	316.2	0.1
P53E	315.8	−0.3
P53 V	315.8	−0.3
R57T	314.9	−1.2
M134Q	314.7	−1.4

^a^The $\widetilde{rc}_{{{\text{ij, }}neighbor}}$ values were converted to a temperature scale according to equation 4 in Ref. [46]

^b^Difference of $\widetilde{rc}_{{{\text{ij, }}neighbor}}$ values of LipA variants minus wtLipA, respectively

### Combination of single amino acid substitutions

Single beneficial amino acid substitutions with different effects were combined to analyze putative synergistic effects on extracellular lipase activity and amount, or additive effects at the level of extracellular lipase amount. To do so, single amino acid substitutions with an increasing effect on either activity (G13S) or amount (A105N and Y139T) were chosen (Fig. 3; Additional file 1: Table S4), and double mutants were generated by site-directed mutagenesis. The corresponding single variants and the wild-type were produced and analyzed again as controls in this experiment confirming the beneficial effects of these substitutions with only slight differences in the absolute numbers.

**Fig. 4:**
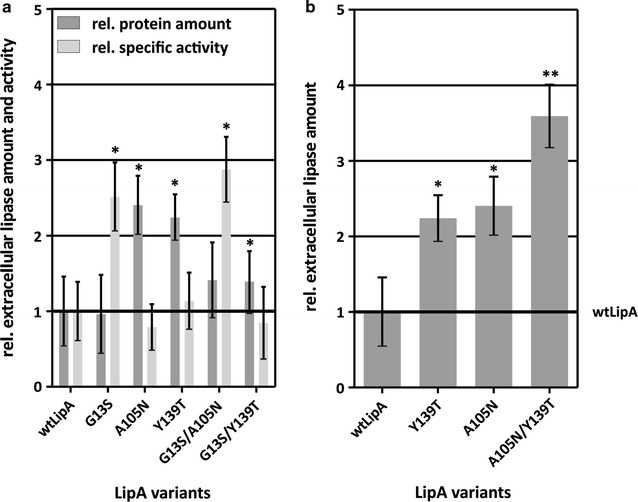
Combination of beneficial single amino acid mutations in LipA and effects on (**a**) extracellular lipase activity and amount, and (**b**) additive effect of two amino acid substitutions on amount of extracellular lipase. *B. subtilis* TEB1030 harboring LipA variants were cultivated for 6 h in a 48 well Flowerplate^®^, and the relative extracellular specific lipase activity (U/mg) in the culture supernatant was calculated by normalizing the volume activity (U/ml) to the protein amount (mg/ml). The extracellular lipase activity was measured using *p*NPP as a substrate, and the extracellular protein amount was determined by ELISA using a specific polyclonal LipA antibody. The relative extracellular lipase amount (dark grey bars) and relative extracellular specific lipase activity (light grey bars) are given relative to wtLipA. Respective wtLipA values were set to 1 (thick black line). Significant changes (*p* < 0.05) compared to wtLipA are marked with a *, significant changes compared to the single variants (*p* < 0.05) are marked with **

No synergistic effect was observed when combining G13S with either A105N or Y139T (Additional file 1: Table S4). When G13S was combined with A105N, the extracellular specific lipase activity of the double mutant G13S/A105N was significantly increased by 2.9-fold compared to wtLipA with 42.7 ± 9.1 U/mg (Fig. 4a; Additional file 1: Table S4), reaching similar levels as the G13S variant. However, the extracellular lipase amount of this double mutant was only slightly increased compared to wtLipA but reduced compared to the A105N variant. The *lipA* transcript amount of the double mutant is not significantly changed compared to wtLipA (Additional file 1: Table S4). This indicates that the G13S substitution, affecting the extracellular lipase activity, largely abolishes the influence of the A105N substitution on protein amount.

The second double mutant G13S/Y139T was unaffected on the level of extracellular specific lipase activity (Fig. 4a; Additional file 1: Table S4) compared to wtLipA and 2.5-fold reduced compared to the G13S single variant. The extracellular lipase amount was 1.4-fold increased compared to wtLipA at similar levels of *lipA* transcript amount, but reduced compared to the single A105N variant (Fig. 4a; Additional file 1: Table S4). Here, both beneficial single amino acid substitutions compensate each other, thus preventing a synergistic beneficial effect when being combined.

However, we also observed an additive effect of two beneficial single mutations in LipA. Variants A105N and Y139T showed a significant increase in extracellular LipA amount of up to 2.4-fold compared to wtLipA with 3.5 ± 0.8 µg/ml at similar levels of extracellular specific lipase activity and similar levels of *lipA* transcript amount (Fig. 4b; Additional file 1: Table S4). The corresponding double mutant LipA A105N/Y139T showed a significant 3.6-fold increase in extracellular LipA amount compared to wtLipA as well as a significant increase of 1.2-fold when compared to the LipA single variants (Fig. 4b; Additional file 1: Table S4).

## Discussion

In this study, we have interrogated the role of single amino acid substitutions of the extracellular lipase LipA from *B. subtilis* with respect to increasing the activity and amount of secreted enzyme. LipA consists of 181 amino acids of which 26 were identified as strictly conserved in 64 lipase sequences within the *Firmicutes* phylum. The remaining 155 amino acids, which are less than 95% conserved, were subjected to a complete site saturation mutagenesis resulting in a library of about 30,000 clones. This library was analyzed to identify clones producing LipA with an increased extracellular activity or an increased amount of lipase protein. The plasmid-based *lipA* expression system increased the extracellular lipase activity from about 0.02 U/ml for the wild-type strain *B. subtilis* 168 [47] to ca. 0.6 U/ml with LipA yields at a mg/l-scale. This is below the g/l-yields obtained under optimized production conditions reported in literature [1, 2], however, it allows measurements also of small effects caused by beneficial substitutions.

### Codon-specific effects

Several LipA variants seem to be affected by the changed codon, but not by the changed amino acid, namely I12L_CTG_, I12V_GTG_, G13T_ACC_, and I87I. A codon substitution can obviously result in a changed amino acid, but can also alter the amount of mRNA, change the transcription rate or the transcript stability as well as the co-translational folding of a protein. We have performed RT-qPCRs to determine the amount of *lipA* transcripts. A mean transcript level of 33 biological and two systematic replicates of wtLipA were calculated resulting in a standard error ranging from 0.4 to 2.2 with the mean value arbitrarily set to 1. Only variants with a changed transcript level below or above this standard error range were assumed to be significantly changed and are discussed here.

An increased amount of transcript may result in an increased protein amount in the supernatant as observed for variant G13T_ACG_ (Fig. 3b; Additional file 1: Table S4) whereas the specific activity remained unaffected. However, the synonymous amino acid substitution in G13T_ACC_ interestingly did not affect the transcript amount but increased the specific activity. Since the same amino acid is introduced, the effect must be caused by the substituted T_ACC_ codon, which is less frequent than the wtLipA codon and the above mentioned T_ACG_ codon (Additional file 1: Table S4). Rare codons can decelerate the translation velocity, that way enabling a more efficient folding of the protein [48], which may explain the increased specific activity of G13T_ACC_. Contrarily, variant I87I also showed an increased specific activity although it contains a more frequent codon (Fig. 3a; Additional file 1: Table S4). The impact of the introduced codon is also illustrated by different I12 variants (Fig. 3a; Additional file 1: Table S4).

### Amino acid substitutions within and near the oxyanion hole can increase specific lipase activity

Five out of the six identified amino acid substitutions increasing extracellular specific lipase activity are located at position 12, forming part of the oxyanion hole [17], or nearby at position 13 (Fig. 3a; Additional file 1: Table S4). This supports former suggestions [49] that optimization approaches should focus on mutations near the substrate-binding site. Substitution of isoleucine by the larger aromatic phenylalanine in variant I12F could lead to a local conformational change, thereby shifting the NH group of the residue at position 12, which could improve the stabilization of the transition state and cause the observed twofold increase in specific activity. Surprisingly, we did not identify substitutions at position M78, the other amino acid forming part of the oxyanion hole [17]. In contrast to I12 and G13, which are located in a flexible turn of LipA, M78 is located in the αC-helix [17]. It is thus possible that substitutions in the αC-helix do not have an effect on LipA activity because conformational changes are sterically hindered. The substitution of glycine with serine in the G13S variant could also lead to a local structural change of LipA in the oxyanion hole region and/or stabilize this region by potential hydrogen bond interactions between the side chains of S13 and R44, that way positively affecting the stabilization of the transition state, which could explain the 1.4-fold increase in specific activity (Fig. 3a; Additional file 1: Table S4).

### Amino acid substitutions improving LipA secretion and stability

In total, 21 LipA variants were identified with amino acid substitutions increasing extracellular LipA amount up to twofold. Six of these variants carry substitutions within the αB-helix of LipA (N50D, P53D, P53E, P53V, R57T_ACC_ and R57T_ACG_; Fig. 3b; Additional file 1: Table S4). Amino acid positions in this helix are known to contribute to detergent tolerance, when substituted to amino acids with charges opposite to the tested detergent [25], and to ionic liquid resistance, when charged and/or polar residues are introduced [45]. Therefore, it is possible that the higher extracellular LipA amount of these variants is not due to a more efficient secretion, but due to an increased stability in the culture supernatant of *B. subtilis*. This stability issue could also underlie the twofold higher extracellular LipA amount of variant M134Q (Fig. 3b; Additional file 1: Table S4). To probe this hypothesis, differences in the thermodynamic thermostability of the LipA variants with respect to wtLipA were predicted by thermal unfolding simulations using CNA; this approach has been previously applied successfully to retro- and prospectively analyze the thermodynamic thermostability of LipA variants [39, 43]. While for three variants (P53D, P53E, P53V) marginal changes in the predicted thermostability compared to wtLipA were found, a pronounced decrease in the thermostability was predicted for the other three variants (N50D, R57T, M134Q). The magnitude of this decrease is in the same ballpark as the magnitude of the median increase in the melting temperature found for 93 cases of engineered proteins, most of which contain more than one mutation [50]. Thus, the results of the CNA analyses do not support the hypothesis that increased *thermodynamic* thermostability of the six variants led to a higher LipA amount in the culture supernatant of *B. subtilis*. However, it should be noted that CNA does not consider time-dependency of processes; hence, our analyses do not rule out an increase in *kinetic* thermostability as a cause for higher extracellular LipA amount.

For the 13 LipA variants I12F, F17E, N48Q, I87V, K88K, A105N, M134K, M134P, Y139G, Y139T, L140A, L140Y, and V154E (Fig. 3b; Additional file 1: Table S4) no stabilizing effects have been described in literature so far. Noteworthy exceptions are amino acid positions N48 and A105, which have been previously identified during thermal unfolding simulations by CNA as structural ‘weak spots’, where mutations could particularly enhance LipA’s thermostability [39].

The identified amino acid positions affecting extracellular protein amount are located in the N- (12, 17, 48), the middle (87, 88, 105), and the C- (134, 139, 140, 154) terminal part of LipA and show no preference regarding the charge of the introduced amino acid. Such randomly distributed mutations within the mature part of an enzyme can affect its secretion as shown for a lipase from *Pseudomonas aeruginosa* [15]. Furthermore, it was demonstrated that N-terminally located amino acids of the mature LamB protein are required for efficient transport in *E. coli* [51]. This could also explain the effect of the three substitutions I12F, F17E, and N48Q in the N-terminal part of LipA. The substitutions identified within the middle (I87V, K88K, A105N) and the C-terminal part of LipA (M134K, M134P, Y139G, Y139T, L140A, L140Y, and V154E) may confer a higher affinity to or allow for a better interaction with components of the translocation machinery such as Sec ATPase or SecYEG translocon [7–10].

### Rational combination of LipA substitutions

In order to answer the question whether a synergistic effect can be achieved by combining single amino acid substitutions that themselves have led to increased specific activity or protein amount, we chose a single amino acid substitution beneficial for extracellular specific lipase activity (G13S; Fig. 3a; Additional file 1: Table S4) and two single amino acid substitutions increasing the extracellular lipase amount (A105N and Y139T; Fig. 3b; Additional file 1: Table S4). The combination of substitutions G13S/A105N and G13S/Y139T (Fig. 4a; Additional file 1: Table S4) resulted in either improved activity, or the effects of the single mutations were abrogated resulting in wild-type level specific activity and protein amount. Apparently, a beneficial mutation can affect e.g. RNA or protein structure or stability. Such effects may thus reinforce or neutralize each other when combined in a double mutant. However, the combination of amino acid substitutions A105N and Y139T, which both individually increased the extracellular protein amount 1.4-fold, resulted in a further increase to 3.6-fold in extracellular protein as compared to the single variants (Fig. 4b; Additional file 1: Table S4), demonstrating in this case an additive effect. Similar additive effects were already described for amino acid substitutions improving thermostability, where 12 amino acid substitutions were introduced by several rounds of in vitro evolution resulting in an increase of the LipA temperature optimum by ~ 30 °C [52]. It should be mentioned that many of such combination experiments need to be carried out before a general conclusion can be drawn.

## Conclusions

In this study, we have systematically analyzed the role of single amino acid and codon substitutions for the secretory production of the model protein LipA in *B. subtilis.* In addition to single amino acid substitutions increasing LipA specific activity and protein amount, we also observed multiple codon-related effects on *lipA* transcription which apparently also influence LipA specific activity. We have identified six LipA variants with increased extracellular specific lipase activity (I12F, I12L_CTG_, I12V_GTG_, G13S, G13T_ACC_, and I87I), of which one also showed an increased extracellular lipase amount (I12F), and a double mutant (A105N/Y139T) which showed an additive effect of the single mutations on the level of extracellular protein amount. The fact that silent mutations can alter the LipA translation rate and thus promote more or less efficient LipA folding is expected to contribute to discussions on the importance of codon bias and abundance in *B. subtilis*, as previously remarked [53]. In summary, we have identified 26 in about 30,000 LipA variants that showed an increase in either amount or specific activity of extracellular lipase. The low success rate and the fact that the most pronounced increases were about twofold only indicate that nature has already optimized production and secretion very well for this lipase in *B. subtilis*. Nevertheless, our results also suggest that optimization campaigns aiming at increased enzyme production may also consider the target protein itself. Variant generation with improved properties might be particularly successful if prioritized towards ‘sensitive’ structural elements, as we find that mutations in the vicinity of the active site on the αB-helix, or at structural ‘weak spots’ showed a higher propensity for improved protein amount and/or activity.

## Additional files

**Additional file 1.** Additional tables.

**Additional file 2.** Additional methods.

**Additional file 3.** Additional figures.
