# Supplementary material for: Contribution of single amino acid and codon substitutions to the production and secretion of a lipase by Bacillus subtilis
Source: Microb Cell Fact. 2017 Sep 25;16:160. doi: 10.1186/s12934-017-0772-z (PMC5613506; doi:10.1186/s12934-017-0772-z)
Supplement: Supplementary file 1 — Additional file 1. Additional tables. [file 12934_2017_772_MOESM1_ESM.doc]

**Additional Tables**

##

## Table S1 Oligonucleotide sequences for generation of LipA site saturation mutagenesis library

The forward and reverse oligonucleotide sequence is shown for each mutated codon positions. Codon positions highlighted in light grey coding for amino acids with a conservation ≥ 95 % (among the Firmicutes) and were not considered for mutagenesis.

| **position** | **forward primer** | **reverse primer** |
| --- | --- | --- |
| 1 | GACTGGATTGTGTTCSNNGGCTTTTGCTGACGG | CCGTCAGCAAAAGCCNNSGAACACAATCCAGTC |
| 2 | AACGACTGGATTGTGSNNAGCGGCTTTTGCTGA | TCAGCAAAAGCCGCTNNSCACAATCCAGTCGTT |
| 3 | CATAACGACTGGATTSNNTTCAGCGGCTTTTGC | GCAAAAGCCGCTGAANNSAATCCAGTCGTTATG |
| 4 | AACCATAACGACTGGSNNGTGTTCAGCGGCTTT | AAAGCCGCTGAACACNNSCCAGTCGTTATGGTT |
| 5 | - | - |
| 6 | - | - |
| 7 | AATACCGTGAACCATSNNGACTGGATTGTGTTC | GAACACAATCCAGTCNNSATGGTTCACGGTATT |
| 8 | TCCAATACCGTGAACSNNAACGACTGGATTGTG | CACAATCCAGTCGTTNNSGTTCACGGTATTGGA |
| 9 | - | - |
| 10 | - | - |
| 11 | - | - |
| 12 | GAATGATGCCCCTCCSNNACCGTGAACCATAAC | GTTATGGTTCACGGTNNSGGAGGGGCATCATTC |
| 13 | ATTGAATGATGCCCCSNNAATACCGTGAACCATAAC | GTTATGGTTCACGGTATTNNSGGGGCATCATTCAAT |
| 14 | AAAATTGAATGATGCSNNTCCAATACCGTGAAC | GTTCACGGTATTGGANNSGCATCATTCAATTTT |
| 15 | CGCAAAATTGAATGASNNCCCTCCAATACCGTG | CACGGTATTGGAGGGNNSTCATTCAATTTTGCG |
| 16 | TCCCGCAAAATTGAASNNTGCCCCTCCAATACC | GGTATTGGAGGGGCANNSTTCAATTTTGCGGGA |
| 17 | AATTCCCGCAAAATTSNNTGATGCCCCTCCAATAC | GTATTGGAGGGGCATCANNSAATTTTGCGGGAATT |
| 18 | CTTAATTCCCGCAAASNNGAATGATGCCCCTCC | GGAGGGGCATCATTCNNSTTTGCGGGAATTAAG |
| 19 | GCTCTTAATTCCCGCSNNATTGAATGATGCCCC | GGGGCATCATTCAATNNSGCGGGAATTAAGAGC |
| 20 | GCTCTTAATTCCSNNAAAATTGAATGATGC | GCATCATTCAATTTTNNSGGAATTAAGAGC |
| 21 | ATAGCTCTTAATSNNCGCAAAATTGAATGATGC | GCATCATTCAATTTTGCGNNSATTAAGAGCTAT |
| 22 | - | - |
| 23 | AGATACGAGATAGCTSNNAATTCCCGCAAAATTG | CAATTTTGCGGGAATTNNSAGCTATCTCGTATCT |
| 24 | CTGAGATACGAGATASNNCTTAATTCCCGC | GCGGGAATTAAGNNSTATCTCGTATCTCAG |
| 25 | GCCCTGAGATACGAGSNNGCTCTTAATTCCCGC | GCGGGAATTAAGAGCNNSCTCGTATCTCAGGGC |
| 26 | CCAGCCCTGAGATACSNNATAGCTCTTAATTCC | GGAATTAAGAGCTATNNSGTATCTCAGGGCTGG |
| 27 | CGACCAGCCCTGAGASNNGAGATAGCTCTTAATTC | GAATTAAGAGCTATCTCNNSTCTCAGGGCTGGTCG |
| 28 | CCGCGACCAGCCCTGSNNTACGAGATAGCTC | GAGCTATCTCGTANNSCAGGGCTGGTCGCGG |
| 29 | GTCCCGCGACCAGCCSNNAGATACGAGATAGCT | AGCTATCTCGTATCTNNSGGCTGGTCGCGGGAC |
| 30 | CTTGTCCCTCGACCASNNCTGAGATACGAGATAG | CTATCTCGTATCTCAGNNSTGGTCGAGGGACAAG |
| 31 | CAGCTTGTCCCGCGASNNGCCCTGAGATACGAG | CTCGTATCTCAGGGCNNSTCGCGGGACAAGCTG |
| 32 | ATACAGCTTGTCCCGSNNCCAGCCCTGAGATAC | GTATCTCAGGGCTGGNNSCGGGACAAGCTGTAT |
| 33 | TGCATACAGCTTGTCSNNCGACCAGCCCTGAG | CTCAGGGCTGGTCGNNSGACAAGCTGTATGCA |
| 34 | AACTGCATACAGCTTSNNCCGCGACCAGCCCTG | CAGGGCTGGTCGCGGNNSAAGCTGTATGCAGTT |
| 35 | ATCAACTGCATACAGSNNGTCCCGCGACCAGCC | GGCTGGTCGCGGGACNNSCTGTATGCAGTTGAT |
| 36 | AAAATCAACTGCATASNNCTTGTCCCGCGACCAG | CTGGTCGCGGGACAAGNNSTATGCAGTTGATTTT |
| 37 | CCAAAAATCAACTGCSNNCAGCTTGTCCCGCGAC | GTCGCGGGACAAGCTGNNSGCAGTTGATTTTTGG |
| 38 | - | - |
| 39 | CTTGTCCCAAAAATCSNNTGCATACAGCTTGTC | GACAAGCTGTATGCANNSGATTTTTGGGACAAG |
| 40 | TGTCTTGTCCCAAAASNNAACTGCATACAGCTT | AAGCTGTATGCAGTTNNSTTTTGGGACAAGACA |
| 41 | GCCTGTCTTGTCCCSNNATCAACTGCATACAG | CTGTATGCAGTTGATNNSGGGACAAGACAGGC |
| 42 | TGTGCCTGTCTTGTCSNNAAAATCAACTGCATA | TATGCAGTTGATTTTNNSGACAAGACAGGCACA |
| 43 | ATTTGTGCCTGTCTTSNNCCAAAAATCAACTGC | GCAGTTGATTTTTGGNNSAAGACAGGCACAAAT |
| 44 | ATAATTTGTGCCTGTSNNGTCCCAAAAATCAAC | GTTGATTTTTGGGACNNSACAGGCACAAATTAT |
| 45 | GTTATAATTTGTGCCSNNCTTGTCCCAAAAATC | GATTTTTGGGACAAGNNSGGCACAAATTATAAC |
| 46 | - | - |
| 47 | TCCATTGTTATAATTSNNGCCTGTCTTGTCCCAAAAATC | GATTTTTGGGACAAGACAGGCNNSAATTATAACAATGGA |
| 48 | CGGTCCATTGTTATASNNTGTGCCTGTCTTGTC | GACAAGACAGGCACANNSTATAACAATGGACCG |
| 49 | TACCGGTCCATTGTTSNNATTTTGTGCCTGTCTT | AAGACAGGCACAAAATNNSAACAATGGACCGGTA |
| 50 | TAATACCGGTCCATTSNNATAATTTGTGCCTGT | ACAGGCACAAATTATNNSAATGGACCGGTATTA |
| 51 | TGATAATACCGGTCCSNNGTTATAATTTGTGCC | GGCACAAATTATAACNNSGGACCGGTATTATCA |
| 52 | TCGTGATAATACCGGSNNATTGTTATAATTTGTG | CACAAATTATAACAATNNSCCGGTATTATCACGA |
| 53 | AAATCGTGATAATACSNNTCCATTGTTATAATT | AATTATAACAATGGANNSGTATTATCACGATTT |
| 54 | CACAAATCGTGATAASNNCGGTCCATTGTTATA | TATAACAATGGACCGNNSTTATCACGATTTGTG |
| 55 | TTGCACAAATCGTGASNNTACCGGTCCATTG | CAATGGACCGGTANNSTCACGATTTGTGCAA |
| 56 | CTTTTGCACAAATCGSNNTAATACCGGTCCATTG | CAATGGACCGGTATTANNSCGATTTGTGCAAAAG |
| 57 | AACCTTTTGCACAAASNNTGATAATACCGGTCC | GGACCGGTATTATCANNSTTTGTGCAAAAGGTT |
| 58 | TAAAACCTTTTGCACSNNTCGTGATAATACCGG | CCGGTATTATCACGANNSGTGCAAAAGGTTTTA |
| 59 | ATCTAAAACCTTTTGSNNAAATCGTGATAATAC | GTATTATCACGATTTNNSCAAAAGGTTTTAGAT |
| 60 | TTCATCTAAAACCTTSNNCACAAATCGATAA | TTATCGATTTGTGNNSAAGGTTTTAGATGAA |
| 61 | CGTTCCATCTAAAACSNNTTGCACAAATCGTG | CACGATTTGTGCAANNSGTTTTAGATGGAACG |
| 62 | - | - |
| 63 | CGCACCCGTTTCATCSNNAACCTTTTGCACAAATC | GATTTGTGCAAAAGGTTNNSGATGAAACGGGTGCG |
| 64 | TTTCGCACCCGTTTCSNNTAAAACCTTTTGCAC | GTGCAAAAGGTTTTANNSGAAACGGGTGCGAAA |
| 65 | TTTTTTCGCACCCGTSNNATCTAAAACCTTTTG | CAAAAGGTTTTAGATNNSACGGGTGCGAAAAAA |
| 66 | - | - |
| 67 | - | - |
| 68 | AATATCCACTTTTTTSNNACCCGTTTCATCTAAAAC | GTTTTAGATGAAACGGGTNNSAAAAAAGTGGATATT |
| 69 | GACAATATCCACTTTSNNCGCACCCGTTTCATC | GATGAAACGGGTGCGNNSAAAGTGGATATTGTC |
| 70 | - | - |
| 71 | - | - |
| 72 | GCTGTGAGCGACAATSNNCACTTTTTTCGCACC | GGTGCGAAAAAAGTGNNSATTGTCGCTCACAGC |
| 73 | - | - |
| 74 | CCCCATGCTGTGAGCSNNAATATCCACTTTTTTC | GAAAAAAGTGGATATTNNSGCTCACAGCATGGGG |
| 75 | - | - |
| 76 | - | - |
| 77 | - | - |
| 78 | TGTGTTCGCGCCCCCSNNGCTGTGAGCGACAATATC | GATATTGTCGCTCACAGCNNSGGGGGCGCGAACACA |
| 79 | - | - |
| 80 | GTAAAGTGTGTTCGCSNNCCCCATGCTGTGAGC | GCTCACAGCATGGGGNNSGCGAACACACTTTAC |
| 81 | GTAGTAAAGTGTGTTSNNGCCCCCCATGCTGTG | CACAGCATGGGGGGCNNSAACACACTTTACTAC |
| 82 | TATGTAGTAAAGTGTSNNCGCGCCCCCCATGCTG | CAGCATGGGGGGCGCGNNSACACTTTACTACATA |
| 83 | TTTTATGTAGTAAAGSNNGTTCGCGCCCCCCATG | CATGGGGGGCGCGAACNNSCTTTACTACATAAAA |
| 84 | ATTTTTTATGTAGTASNNTATGTTCGCGCCCCC | GGGGGCGCGAACATANNSTACTACATAAAAAAT |
| 85 | CAGATTTTTTATGTASNNAAGTGTGTTCGCGCC | GGCGCGAACACACTTNNSTACATAAAAAATCTG |
| 86 | - | - |
| 87 | GCCGTCCAGATTTTTSNNGTAGTAAAGTGTGTTC | GAACACACTTTACTACNNSAAAAATCTGGACGGC |
| 88 | TCCGCCGTCCAGATTSNNTATGTAGTAAAGTGTG | CACACTTTACTACATANNSAATCTGGACGGCGGA |
| 89 | ATTTCCGCCGTCCAGSNNTTTTATGTAGTAAAG | CTTTACTACATAAAANNSCTGGACGGCGGAAAT |
| 90 | TTTATTTCCGCCGTCSNNATTTTTTATGTAGTAAAG | CTTTACTACATAAAAAATNNSGACGGCGGAAATAAA |
| 91 | AACTTTATTTCCGCCSNNCAGATTTTTTATGTAG | CTACATAAAAAATCTGNNSGGCGGAAATAAAGTT |
| 92 | TGCAACTTTATTTCCSNNGTCCAGATTTTTTATG | CATAAAAAATCTGGACNNSGGAAATAAAGTTGCA |
| 93 | GTTTGCAACTTTATTSNNGCCGTCCAGATTTTTTATG | CATAAAAAATCTGGACGGCNNSAATAAAGTTGCAAAC |
| 94 | GACGTTTGCAACTTTSNNTCCGCCGTCCAGATTTTTTATG | CATAAAAAATCTGGACGGCGGANNSAAAGTTGCAAACGTC |
| 95 | CACGACGTTTGCAACSNNATTTCCGCCGTCCAG | CTGGACGGCGGAAATNNSGTTGCAAACGTCGTG |
| 96 | CGTCACGACGTTTGCSNNTTTATTTCCGCCGTC | GACGGCGGAAATAAANNSGCAAACGTCGTGACG |
| 97 | AAGCGTCACGACGTTSNNAACTTTATTCCTGCC | GGCAGGAATAAAGTTNNSAACGTCGTGACGCTT |
| 98 | GCCAAGCGTCACGACSNNTGCAACTTTATTTCC | GGAAATAAAGTTGCANNSGTCGTGACGCTTGGC |
| 99 | GCCGCCAAGCGTCACSNNGTTTGCAACTTTATTTC | GAAATAAAGTTGCAAACNNSGTGACGCTTGGCGGC |
| 100 | CGCGCCGCCAAGCGTSNNGACGTTTGCAAC | GTTGCAAACGTCNNSACGCTTGGCGGCGCG |
| 101 | GTTCGCGCCGCCAAGSNNCACGACGTTTGCAAC | GTTGCAAACGTCGTGNNSCTTGGCGGCGCGAAC |
| 102 | ACGGTTCGCGCCGCCSNNCGTCACGACGTTTGC | GCAAACGTCGTGACGNNSGGCGGCGCGAACCGT |
| 103 | - | - |
| 104 | CGTTAAACGGTTCGCSNNGCCAAGCGTCACGAC | GTCGTGACGCTTGGCNNSGCGAACCGTTTAACG |
| 105 | TGTCGTTAAACGGTTSNNGCCGCCAAGCGTCAC | GTGACGCTTGGCGGCNNSAACCGTTTAACGACA |
| 106 | - | - |
| 107 | CTTGCCTGTCGTCAASNNGTTCGCGCCGCCAGG | CCTGGCGGCGCGAACNNSTTGACGACAGGCAAG |
| 108 | CGCCTTGCCTGTCGTSNNACGGTTCGCGCCGCC | GGCGGCGCGAACCGTNNSACGACAGGCAAGGCG |
| 109 | AAGCGCCTTGCCTGTSNNCAAACGGTTCGCGCC | GGCGCGAACCGTTTGNNSACAGGCAAGGCGCTT |
| 110 | CCGAAGCGCCTTGCCSNNCGTCAAACGGTTCGC | GCGAACCGTTTGACGNNSGGCAAGGCGCTTCGG |
| 111 | CCCGGAAGCGCCTTSNNTGTCGTCAAACGGTTC | GAACCGTTTGACGACANNSAAGGCGCTTCCGGG |
| 112 | TGTTCCCCGAAGCGCSNNGCCTGTCGTCAAACG | CGTTTGACGACAGGCNNSGCGCTTCGGGGAACA |
| 113 | ATCTGTTCCCGGAAGSNNCTTGCCTGTCGTTAAAC | GTTTAACGACAGGCAAGNNSCTTCCGGGAACAGAT |
| 114 | TGGATCTGTTCCCGGSNNCGCCTTGCCTGTCG | CGACAGGCAAGGCGNNSCCGGGAACAGATCCA |
| 115 | ATTTGGATCTGTTCCSNNAAGCGCCTTGCCTGTC | GACAGGCAAGGCGCTTNNSGGAACAGATCCAAAT |
| 116 | TTGATTTGGATCTGTSNNCGGAAGCGCCTTGCC | GGCAAGGCGCTTCCGNNSACAGATCCAAATCAA |
| 117 | CTTTTGATTTGGATCSNNTCCCGGAAGCGCCTTG | CAAGGCGCTTCCGGGANNSGATCCAAATCAAAAG |
| 118 | AATCTTTTGATTTGGSNNTGTTCCCGGAAGCGC | GCGCTTCCGGGAACANNSCCAAATCAAAAGATT |
| 119 | TAAAATCTTTTGATTSNNATCTGTTCCCGGAAG | CTTCCGGGAACAGATNNSAATCAAAAGATTTTA |
| 120 | GTATAAAATCTTTTGSNNTGGATCTGTTCCCGG | CCGGGAACAGATCCANNSCAAAAGATTTTATAC |
| 121 | TGTGTATAAAATCTTSNNATTTGGATCTGTTCC | GGAACAGATCCAAATNNSAAGATTTTATACACA |
| 122 | GGATGTGTATAAAATSNNTTGATTTGGATCTG | CAGATCCAAATCAANNSATTTTATACACATCC |
| 123 | AATGGATGTGTATAASNNCTTTTGATTTGGATC | GATCCAAATCAAAAGNNSTTATACACATCCATT |
| 124 | GTAAATGGATGTGTASNNATTCTTTTGATTTGG | CCAAATCAAAAGAATNNSTACACATCCATTTAC |
| 125 | GCTGTAAATGGATGTSNNTAAAATCTTTTGATTTG | CAAATCAAAAGATTTTANNSACATCCATTTACAGC |
| 126 | ACTGCTGTAAATGGASNNGTATAAAATCTTTTG | CAAAAGATTTTATACNNSTCCATTTACAGCAGT |
| 127 | GGCACTGCTGTAAATSNNTGTGTATAAAATCTT | AAGATTTTATACACANNSATTTACAGCAGTGCC |
| 128 | ATCGGCACTGCTGTASNNGGATGTGTATAAAATC | GATTTTATACACATCCNNSTACAGCAGTGCCGAT |
| 129 | - | - |
| 130 | AATCATATCGGCACTSNNGTAAATGGATGTG | CACATCCATTTACNNSAGTGCCGATATGATT |
| 131 | GACAATCATATCGGCSNNGCTGTAAATGGATG | CATCCATTTACAGCNNSGCCGATATGATTGTC |
| 132 | CATGACAATCATATCSNNACTGCTGTAAATGG | CCATTTACAGCAGTNNSGATATGATTGTCATG |
| 133 | ATTCATGACAATCATSNNGGCACTGCTGTAAATG | CATTTACAGCAGTGCCNNSATGATTGTCATGAAT |
| 134 | GTAATTCATGACAATSNNATCGGCACTGCTGTAAATG | CATTTACAGCAGTGCCGATNNSATTGTCATGAATTAC |
| 135 | TAAGTAATTCATGACSNNCATATCGGCACTGCTG | CAGCAGTGCCGATATGNNSGTCATGAATTACTTA |
| 136 | TGATAAGTAATTCATSNNAATCATATCGGCACTG | CAGTGCCGATATGATTNNSATGAATTACTTATCA |
| 137 | TCTTGATAAGTAATTSNNGACAATCATATCGGC | GCCGATATGATTGTCNNSAATTACTTATCAAGA |
| 138 | TAATCTTGATAAGTASNNCATGACAATCATATC | GATATGATTGTCATGNNSTACTTATCAAGATTA |
| 139 | ATCTAATCTTGATAASNNATTCATGACAATCATATCGGC | GCCGATATGATTGTCATGAATNNSTTATCAAGATTAGAT |
| 140 | ACCATCTAATCTTGASNNGTAATTCATGACAATC | GATTGTCATGAATTACNNSTCAAGATTAGATGGT |
| 141 | - | - |
| 142 | TCTAGCACCATCTAASNNTGATAAGTAATTCATG | CATGAATTACTTATCANNSTTAGATGGTGCTAGA |
| 143 | GTTTCTAGCACCATCSNNTCTTGATAAGTAATTC | GAATTACTTATCAAGANNSGATGGTGCTAGAAAC |
| 144 | AACGTTTCTAGCACCSNNTAATCTTGATAAGTAATT | AATTACTTATCAAGATTANNSGGTGCTAGAAACGTT |
| 145 | - | - |
| 146 | GATTTGAACGTTTCTSNNACCATCTAATCTTG | CAAGATTAGATGGTNNSAGAAACGTTCAAATC |
| 147 | ATGGATTTGAACGTTSNNAGCACCATCTAATCTTG | CAAGATTAGATGGTGCTNNSAACGTTCAAATCCAT |
| 148 | GCCATGGATTTGAACSNNTCTAGCACCATCTAATC | GATTAGATGGTGCTAGANNSGTTCAAATCCATGGC |
| 149 | AACGCCATCGATTTGSNNGTTTCTAGCACCATC | GATGGTGCTAGAAACNNSCAAATCGATGGCGTT |
| 150 | TCCAACGCCATGGATSNNAACGTTTCTAGCACC | GGTGCTAGAAACGTTNNSATCCATGGCGTTGGA |
| 151 | GTGTCCAACGCCATGSNNTTGAACGTTTCTAGC | GCTAGAAACGTTCAANNSCATGGCGTTGGACAC |
| 152 | GATGTGTCCAACGCCSNNGATTTGAACGTTTCTAG | CTAGAAACGTTCAAATCNNSGGCGTTGGACACATC |
| 153 | - | - |
| 154 | AAGGCCGATGTGTCCSNNGCCATGGATTTGAAC | GTTCAAATCCATGGCNNSGGACACATCGGCCTT |
| 155 | CAGAAGGCCGATGTGSNNAACGCCATGGATTTG | CAAATCCATGGCGTTNNSCACATCGGCCTTCTG |
| 156 | GTACAGAAGGCCGATSNNTCCAACGCCATGGATTTG | CAAATCCATGGCGTTGGANNSATCGGCCTTCTGTAC |
| 157 | GCTGTACAGAAGGCCSNNGTGTCCAACGCCATG | CATGGCGTTGGACACNNSGGCCTTCTGTACAGC |
| 158 | GCTGCTGTACAGAAGSNNGATGTGTCCAACGCC | GGCGTTGGACACATCNNSCTTCTGTACAGCAGC |
| 159 | TTGGCTGCTGTACAGSNNGCCGATGTGTCCAAC | GTTGGACACATCGGCNNSCTGTACAGCAGCCAA |
| 160 | GACTTGGCTGCTGTASNNAAGGCCGATGTGTCC | GGACACATCGGCCTTNNSTACAGCAGCCAAGTC |
| 161 | GTTGACTTGGCTGCTSNNCAGAAGGCCGATGTG | CACATCGGCCTTCTGNNSAGCAGCCAAGTCAAC |
| 162 | GCTGTTGACTTGGCTSNNGTACAGAAGGCCGATG | CATCGGCCTTCTGTACNNSAGCCAAGTCAACAGC |
| 163 | CAGGCTGTTGACTTGSNNGCTGTACAGAAGGCC | GGCCTTCTGTACAGCNNSCAAGTCAACAGCCTG |
| 164 | AATCAGGCTGTTGACSNNGCTGCTGTACAGAAG | CTTCTGTACAGCAGCNNSGTCAACAGCCTGATT |
| 165 | - | - |
| 166 | TTCTTTAATCAGGCTSNNGACTTGGCTGCTGTAC | GTACAGCAGCCAAGTCNNSAGCCTGATTAAAGAA |
| 167 | CCCTCCTTTAATCAGSNNGTTGACTTGGCTGCTG | CAGCAGCCAAGTCAACNNSCTGATTAAAGGAGGG |
| 168 | CAGCCCTTCTTTAATSNNGCTGTTGACTTGGCTGCTG | CAGCAGCCAAGTCAACAGCNNSATTAAAGAAGGGCTG |
| 169 | GTTCAGCCCTTCTTTSNNCAGGCTGTTGACTTG | CAAGTCAACAGCCTGNNSAAAGAAGGGCTGAAC |
| 170 | GCCGTTCAGCCCTTCSNNAATCAGGCTGTTGAC | GTCAACAGCCTGATTNNSGAAGGGCTGAACGGC |
| 171 | CCCGCCGTTCAGCCCSNNTTTAATCAGGCTGTT | AACAGCCTGATTAAANNSGGGCTGAACGGCGGG |
| 172 | GCCCCCGCCGTTCAGSNNTTCTTTAATCAGGCT | AGC CTG ATT AAA GAA NNS CTG AAC GGC GGG GGC |
| 173 | CTGGCCCCCGCCGTTSNNCCCTTCTTTAATCAG | CTG ATT AAA GAA GGG NNS AAC GGC GGG GGC CAG |
| 174 | TTCTGGCCCCCGCCSNNCAGCCCTTCTTTAATC | GATTAAAGAAGGGCTGNNSGGCGGGGGCCAGAA |
| 175 | CGTATTCTGGCCCCCSNNGTTCAGCCCTTCTTT | AAA GAA GGG CTG AAC NNS GGG GGC CAG AAT ACG |
| 176 | ATTCGTATTCTGGCCSNNGCCGTTCAGCCCTTC | GAA GGG CTG AAC GGC NNS GGC CAG AAT ACG AAT |
| 177 | TTAATTCGTATTCTGSNNCCCGCCGTTCAGCCC | GGG CTG AAC GGC GGG NNS CAG AAT ACG AAT TAA |
| 178 | GCTTGTCGACGGAGCTCTCATTAATTCGTATTSNNGCC | GGCNNSAATACGAATTAATGAGAGCTCCGTCGACAAGC |
| 179 | GCTTGTCGACGGAGCTCTCATTAATTCGTSNNCTC | GAGNNSACGAATTAATGAGAGCTCCGTCGACAAGC |
| 180 | GCTTGTCGACGGAGCTCTCATTAATTSNNATT | AATNNSAATTAATGAGAGCTCCGTCGACAAGC |
| 181 | GCTTGTCGACGGAGCTCTCATTASNNCGT | ACGNNSTAATGAGAGCTCCGTCGACAAGC |

## Table S2 Oligonucleotide sequences for generation of *lipA* site directed single and double mutants

The forward and reverse oligonucleotide sequence is shown for each variant. Modification sites are underlined.

| **variant** | **forward primer** | **reverse primer** |
| --- | --- | --- |
| G13S | ATGGTTCACGGTATTTCGGGGGCATCATTCAAT | ATTGAATGATGCCCCCGAAATACCGTGAACCAT |
| A105N | GTGACGCTTGGCGGCAACAACCGTTTGACGACA | TGTCGTCAAACGGTTGTTGCCGCCAAGCGTCAC |
| Y139T | ATGATTGTCATGAATACCTTATCAAGATTAGAT | ATCTAATCTTGATAAGGTATTCATGACAATCAT |

## Table S3 LipA amino acid sequence conservation

**A**: UniProtKB accession numbers and original organisms for the 64 lipase sequences out of 41 species from the *Firmicutes* phylum used for the alignment. **B**: The number of identical amino acids in this alignment like in *B. subtilis* LipA was counted and calculated in percentage frequency for each position to determine the conservation of this amino acid within the *Firmicutes* phylum. The amino acid position (position), the amino acid (aa) and the percentaged conservation are shown.

A

| **Lipase** | **Species** | **Lipase** | **Species** | **Lipase** | **Species** | **Lipase** | **Species** |
| --- | --- | --- | --- | --- | --- | --- | --- |
| **P94444** | Bacillus sp. BP-6 | **Q8VU78** | Bacillus sp. B26 | **H0FRJ5** | *Bacillus amyloliquefaciens* *IT-45* | **E0U0Y0** | Bacillus subtilis spizizenii ATCC 23059 |
| **Q79F14** | Bacillus subtilis 168 | **H6U4T6** | Bacillus sp. enrichment culture clone S6 | **H2ABY2** | *Bacillus amyloliquefaciens* subsp. *plantarum* CAU B946 | **G4NTQ6** | Bacillus subtilis spizizenii TU-B-10 |
| **B8YLY0** | Bacillus subtilis | **B2L2K1** | Bacillus licheniformis | **H8XE51** | *Bacillus amyloliquefaciens* subsp. *plantarum* YAU B9601-Y2 | **E5W0L6** | *Bacillus sp.* BT1B_CT2 |
| **Q8RJP5** | Bacillus megaterium | **A1E152** | Bacillus pumilus | **F4E233** | *Bacillus amyloliquefaciens* TA208 | **Q65HR4** | Bacillus licheniformis ATCC 14580 |
| **E8VEC0** | Bacillus subtilis SC-8 | **A8FGA4** | Bacillus pumilus SAFR-032 | **G0IK64** | *Bacillus amyloliquefaciens* XH7 | **I0UHQ4** | *Bacillus licheniformis* WX-02 |
| **G4EYR4** | Bacillus subtilis SC-8 | **Q9K5F4** | Bacillus licheniformis | **E3DTQ6** | Bacillus atrophaeus 1942 | **B3F2Y4** | Bacillus sp. RN2 |
| **G4P2C8** | *Bacillus subtilis* RO-NN-1 | **Q6RSN0** | Bacillus pumilus | **A5HLW9** | Bacillus subtilis | **H6NI24** | Paenibacillus mucilaginosus 3016 |
| **D5N1Z7** | *Bacillus subtilis* subsp. *spizizenii* ATCC 6633 | **B1PN85** | Bacillus pumilus | **G4PA03** | *Bacillus subtilis* RO-NN-1 | **I0BL71** | Paenibacillus mucilaginosus K02 |
| **E0TW96** | *Bacillus subtilis* ATCC 23059 | **B4ANV6** | Bacillus pumilus ATCC 7061 | **B1PN84** | Bacillus subtilis | **F8FBS6** | Paenibacillus mucilaginosus KNP414 |
| **G4NRF1** | *Bacillus subtilis* TU-B-10 | **Q2LAN2** | Bacillus pumilus | **D4G4R9** | Bacillus subtilis subsp. natto BEST195 | **Q5WDN0** | Bacillus clausii KSM-K16 |
| **D4G6J8** | Bacillus subtilis subsp. natto BEST195 | **B7VF67** | Bacillus pumilus | **E8VK85** | *Bacillus subtilis* BSn5 | **Q8RC83** | Caldanaerobacter subterraneus subsp. tengcongensis DSM 15242 |
| **B0LW76** | Bacillus sp. NK13 | **B8Y3H3** | Bacillus pumilus | **G4F0D0** | Bacillus subtilis SC-8 | **Q6WUB2** | Caldanaerobacter subterraneus subsp. tengcongensis |
| **D5E2W8** | Bacillus megaterium ATCC 12872 | **B2CX98** | Bacillus pumilus | **B7UDC5** | Bacillus subtilis | **F1ZT35** | Thermoanaerobacter ethanolicus JW 200 |
| **D3WK98** | Bacillus pumilus | **Q2L991** | Bacillus pumilus | **I0F008** | Bacillus sp. JS | **G2MS56** | Thermoanaerobacter wiegelii Rt8.B1 |
| **E2CYQ9** | Bacillus pumilus | **D7URU5** | Bacillus sp. HH-01 | **Q83ZY1** | Bacillus subtilis | **D3FQU1** | Bacillus pseudofirmus OF4 |
| **A4GUJ6** | Bacillus pumilus | **A7Z124** | Bacillus velezensis DSM 23117 | **D5N2V3** | *Bacillus subtilis* subsp. *spizizenii* ATCC 6633 | **P37957** | Bacillus subtilis 168 |

B

| **position** | **aa** | **%** | **position** | **aa** | **%** | **position** | **aa** | **%** | **position** | **aa** | **%** |
| --- | --- | --- | --- | --- | --- | --- | --- | --- | --- | --- | --- |
| 1 | A | 55 | 52 | G | 66 | 103 | G | 97 | 154 | V | 69 |
| 2 | E | 43 | 53 | P | 85 | 104 | G | 91 | 155 | G | 78 |
| 3 | H | 83 | 54 | V | 20 | 105 | A | 91 | 156 | H | 92 |
| 4 | N | 83 | 55 | L | 92 | 106 | N | 98 | 157 | I | 85 |
| 5 | P | 97 | 56 | S | 65 | 107 | R | 29 | 158 | G | 91 |
| 6 | V | 95 | 57 | R | 48 | 108 | L | 91 | 159 | L | 91 |
| 7 | V | 89 | 58 | F | 52 | 109 | T | 23 | 160 | L | 92 |
| 8 | M | 65 | 59 | V | 80 | 110 | T | 49 | 161 | Y | 11 |
| 9 | V | 98 | 60 | Q | 28 | 111 | G | 15 | 162 | S | 49 |
| 10 | H | 97 | 61 | K | 38 | 112 | K | 23 | 163 | S | 94 |
| 11 | G | 100 | 62 | V | 98 | 113 | A | 89 | 164 | Q | 80 |
| 12 | I | 74 | 63 | L | 92 | 114 | L | 78 | 165 | V | 97 |
| 13 | G | 72 | 64 | D | 35 | 115 | P | 77 | 166 | N | 62 |
| 14 | G | 91 | 65 | E | 52 | 116 | G | 86 | 167 | S | 22 |
| 15 | A | 86 | 66 | T | 97 | 117 | T | 78 | 168 | L | 43 |
| 16 | S | 77 | 67 | G | 98 | 118 | D | 78 | 169 | I | 80 |
| 17 | F | 28 | 68 | A | 94 | 119 | P | 75 | 170 | K | 85 |
| 18 | N | 92 | 69 | K | 72 | 120 | N | 80 | 171 | E | 78 |
| 19 | F | 94 | 70 | K | 97 | 121 | Q | 78 | 172 | G | 85 |
| 20 | A | 62 | 71 | V | 100 | 122 | K | 85 | 173 | L | 92 |
| 21 | G | 38 | 72 | D | 92 | 123 | I | 85 | 174 | N | 75 |
| 22 | I | 97 | 73 | I | 100 | 124 | L | 78 | 175 | G | 75 |
| 23 | K | 89 | 74 | V | 92 | 125 | Y | 92 | 176 | G | 83 |
| 24 | S | 45 | 75 | A | 95 | 126 | T | 92 | 177 | G | 86 |
| 25 | Y | 89 | 76 | H | 98 | 127 | S | 91 | 178 | Q | 55 |
| 26 | L | 94 | 77 | S | 98 | 128 | I | 63 | 179 | N | 78 |
| 27 | V | 48 | 78 | M | 91 | 129 | Y | 97 | 180 | T | 71 |
| 28 | S | 65 | 79 | G | 98 | 130 | S | 91 | 181 | N | 71 |
| 29 | Q | 88 | 80 | G | 94 | 131 | S | 60 |  |  |  |
| 30 | G | 92 | 81 | A | 91 | 132 | A | 57 |  |  |  |
| 31 | W | 89 | 82 | N | 91 | 133 | D | 92 |  |  |  |
| 32 | S | 32 | 83 | T | 86 | 134 | M | 22 |  |  |  |
| 33 | R | 52 | 84 | L | 91 | 135 | I | 91 |  |  |  |
| 34 | D | 22 | 85 | Y | 89 | 136 | V | 91 |  |  |  |
| 35 | K | 43 | 86 | Y | 97 | 137 | M | 26 |  |  |  |
| 36 | L | 78 | 87 | I | 89 | 138 | N | 91 |  |  |  |
| 37 | Y | 77 | 88 | K | 85 | 139 | Y | 26 |  |  |  |
| 38 | A | 97 | 89 | N | 75 | 140 | L | 91 |  |  |  |
| 39 | V | 26 | 90 | L | 89 | 141 | S | 98 |  |  |  |
| 40 | D | 83 | 91 | D | 69 | 142 | R | 77 |  |  |  |
| 41 | F | 92 | 92 | G | 91 | 143 | L | 89 |  |  |  |
| 42 | W | 20 | 93 | G | 86 | 144 | D | 28 |  |  |  |
| 43 | D | 85 | 94 | N | 38 | 145 | G | 97 |  |  |  |
| 44 | K | 88 | 95 | K | 89 | 146 | A | 83 |  |  |  |
| 45 | T | 85 | 96 | V | 34 | 147 | R | 58 |  |  |  |
| 46 | G | 98 | 97 | A | 25 | 148 | N | 89 |  |  |  |
| 47 | T | 20 | 98 | N | 78 | 149 | V | 65 |  |  |  |
| 48 | N | 92 | 99 | V | 86 | 150 | Q | 60 |  |  |  |
| 49 | Y | 28 | 100 | V | 83 | 151 | I | 69 |  |  |  |
| 50 | N | 74 | 101 | T | 89 | 152 | H | 58 |  |  |  |
| 51 | N | 91 | 102 | L | 80 | 153 | G | 97 |  |  |  |

## Table S4 Summary of relative transcript amount, specific activity and protein amount of 38 characterized LipA variants

The table shows the structural position and location of the variant’s amino acid substitution. The wild-type (wt) codon and the introduced variant codon are named together with the codon frequency per 1000 bp. Variant I12F as variant with increased specific activity as well as protein amount is shown twice.

| **false-positive LipA variants** | | | | | | | | | | |
| --- | --- | --- | --- | --- | --- | --- | --- | --- | --- | --- |
| **variant** | **position in secondary structure** | **location** | **wt codon** | **frequency per 1000bp** | **variant codon** | **frequency per 1000bp** | **rel. change in transcript level^2^** | **lower and upper deviation in transcript level** | **rel. specific activity ± standard deviation** | **rel. lipase amount ± standard deviation** |
| I12L_TTG_ | turn | s | ATT | 36.2 | TTG | 15.8 | 1.3 | 0.4 0.7 | 0.8 ± 0.3 | 1.0 ± 0.4 |
| I12V_GTC_ | turn | s | ATT | 36.2 | GTC | 17.3 | 1.4 | 0.4 0.7 | 0.8 ± 0.6 | 1.0 ± 0.3 |
| G13N | turn | s | GGA | 21.8 | AAC | 17.8 | 1 | 0.4 0.6 | 0.7 ± 0.4 | 0.8 ± 0.3 |
| Q29H | turn | s | CAG | 18.5 | CAC | 7.5 | 0.8 | 0.2 0.2 | 0.9 ± 0.4 | 1.0 ± 0.3 |
| T47H | coil | s | ACA | 21.6 | CAC | 7.5 | 0.3 | 0.3 1.0 | 0.9 ± 0.4 | 1.2 ± 0.3 |
| T47P | coil | s | ACA | 21.6 | CCA | 7.4 | 1.5 | 0.4 0.4 | 1 ± 0.3 | 1.0 ± 0.3 |
| T47T | coil | s | ACA | 21.6 | ACG | 14.9 | 1.4 | 0.4 0.6 | 1.0 ± 0.4 | 0.7 ± 0.3 |
| N48G | αB | s | AAT | 22.9 | GGC | 23.3 | 1.2 | 0.2 0.4 | 0.3 ± 0.3 | 1.4 ± 0.3 |
| L55F | αB | b | TTA | 19.8 | TTC | 14.3 | 1 | 0.2 0.2 | 0.4 ± 0.4 | 1.2 ± 0.3 |
| T83M | αC | b | ACA | 21.6 | ATG | 26.3 | 1.5 | 0.3 0.5 | 0.6 ± 0.4 | 0.9 ± 0.4 |
| Y85W | αC | s | TAC | 12.6 | TTG | 15.8 | 1.4 | 0.4 0.8 | 0.7 ± 0.4 | 0.6 ± 0.3 |
| I87L | αC | s | ATA | 9.8 | CTC | 10.7 | 1 | 0.9 6.6 | 0.1 ± 0.4 | 1.1 ± 0.3 |
| **LipA variants with increased extracellular specific lipase activity** | | | | | | | | | | |
| **variant** | **position in secondary structure** | **location** | **wt codon** | **frequency per 1000bp** | **variant codon** | **frequency per 1000bp** | **rel. change in transcript level^2^** | **lower and upper deviation in transcript level** | **rel. specific activity ± standard deviation** | **rel. lipase amount ± standard deviation** |
| I12F | turn | s | ATT | 36.2 | TTC | 14.3 | 1.7 | 0.4 0.5 | 2.1* ± 0.4 | 1.6* ± 0.2 |
| I12L_CTG_ | turn | s | ATT | 36.2 | CTG | 23.0 | 1.0 | 0.4 0.7 | 2.4* ± 0.3 | 0.9 ± 0.3 |
| I12V_GTG_ | turn | s | ATT | 36.2 | GTG | 17.3 | 1.3 | 0.3 0.6 | 1.8* ± 0.4 | 1.0 ± 0.3 |
| G13S | turn | s | GGA | 21.8 | TCG | 6.5 | 1.4 | 0.5 0.5 | 1.4* ± 0.3 | 0.6 ± 0.4 |
| G13T_ACC_ | turn | s | GGA | 21.8 | ACC | 9.0 | 1.2 | 0.4 0.5 | 2.3* ± 0.3 | 0.5 ± 0.2 |
| I87I | αC | s | ATA | 9.8 | ATC | 27.2 | 3.6* | 0.6 0.9 | 2.3* ± 0.3 | 0.7 ± 0.4 |
| **LipA variants with higher extracellular LipA amount** | | | | | | | | | | |
| **variant** | **position in secondary structure** | **location** | **wt codon** | **frequency per 1000bp** | **variant codon** | **frequency per 1000bp** | **rel. change in transcript level^2^** | **lower and upper deviation in transcript level** | **rel. specific activity ± standard deviation** | **rel. lipase amount ± standard deviation** |
| I12F | turn | s | ATT | 36.2 | TTC | 14.3 | 1.7 | 0.4 0.5 | 2.1* ± 0.4 | 1.6* ± 0.2 |
| G13T_ACG_ | turn | s | GGA | 21.8 | ACG | 14.9 | 2.7* | 0.9 1.2 | 0.8 ± 0.4 | 1.6* ± 0.3 |
| F17E | αA | s | TTC | 14.3 | GAG | 22.6 | 2 | 0.6 0.9 | 0.5 ± 0.4 | 1.3* ± 0.3 |
| N48Q | αB | s | AAT | 22.9 | CAG | 18.5 | 2.2 | 0.6 1.2 | 0.6 ± 0.3 | 2.2* ± 0.2 |
| N50D | αB | s | AAC | 17.8 | GAC | 19.0 | 0.8 | 0.3 0.4 | 0.8 ± 0.5 | 1.3* ± 0.3 |
| P53D | αB | s | CCG | 16.3 | GAC | 19.0 | 1.0 | 0.2 0.3 | 0.7 ± 0.4 | 1.9* ± 0.3 |
| P53E | αB | s | CCG | 16.3 | GAG | 22.6 | 1.0 | 0.1 0.2 | 0.9 ± 0.4 | 1.5* ± 0.3 |
| P53V | αB | s | CCG | 16.3 | GTG | 17.3 | 1.3 | 0.3 0.5 | 0.7 ± 0.3 | 1.4* ± 0.3 |
| R57T_ACC_ | αB | s | CGA | 4.3 | ACC | 9.0 | 0.8 | 0.3 0.7 | 0.8 ± 0.4 | 1.5* ± 0.3 |
| R57T_ACG_ | αB | s | CGA | 4.3 | ACG | 14.9 | 0.9 | 0.2 0.3 | 0.7 ± 0.4 | 1.4* ± 0.2 |
| I87V | αC | s | ATA | 9.8 | GTG | 17.3 | 1.3 | 1.2 8.6 | 1.1 ± 0.3 | 1.6* ± 0.3 |
| K88K | αC | s | AAA | 48.4 | AAG | 20.8 | 1.6 | 0.4 0.5 | 0.4 ± 0.3 | 1.4* ± 0.3 |
| A105N | coil | s | GCG | 19.8 | AAC | 17.8 | 1.2 | 0.3 0.3 | 0.5 ± 0.3 | 2.0* ± 0.3 |
| M134K | coil | s | ATG | 26.3 | AAG | 20.8 | 1.1 | 0.3 0.5 | 0.9 ± 0.4 | 2.3* ± 0.3 |
| M134P | coil | s | ATG | 26.3 | CCG | 16.3 | 0.6 | 0.1 0.1 | 0.2 ± 0.3 | 2.5* ± 0.2 |
| M134Q | coil | s | ATG | 26.3 | CAG | 18.5 | 0.8 | 0.2 0.2 | 0.4 ± 0.3 | 2.1* ± 0.3 |
| Y139G | αE | s | TAC | 12.6 | GGG | 11.2 | 1.7 | 0.7 0.8 | 0.6 ± 0.4 | 1.5* ± 0.3 |
| Y139T | αE | s | TAC | 12.6 | ACG | 14.9 | 2.1 | 0.5 0.5 | 0.5 ± 0.4 | 1.8* ± 0.3 |
| L140A | αE | s | TTA | 19.8 | GCG | 19.8 | 1.6 | 0.4 0.5 | 0.4 ± 0.3 | 2.2* ± 0.2 |
| L140Y | αE | s | TTA | 19.8 | TAC | 12.6 | 1.6 | 0.5 1.3 | 0.6 ± 0.4 | 1.8* ± 0.2 |
| V154E | coil | s | GTT | 18.6 | GAG | 22.6 | 0.9 | 0.3 0.4 | 0.6 ± 0.3 | 1.3* ± 0.2 |
| **Combined LipA variants** | | | | | | | | | | |
| **variant** | **position in secondary structure** | **location^1^** | **wt codon** | **frequency per 1000bp** | **variant codon** | **frequency per 1000bp** | **rel. change in transcript level^2^** | **lower and upper deviation in transcript level** | **rel. specific activity ± standard deviation** | **rel. lipase amount ± standard deviation** |
| G13S | turn | s | GGA | 21.8 | TCG | 6.5 | 1.8 | 0.6 1.2 | 2.5* ± 0.5 | 1.0 ± 0.5 |
| A105N | coil | s | GCG | 19.8 | AAC | 17.8 | 2.1 | 0.6 1.3 | 0.8 ± 0.3 | 2.4* ± 0.4 |
| Y139T | αE | s | TAC | 12.6 | ACG | 14.9 | 0.9 | 0.2 0.2 | 1.1 ± 0.4 | 2.2* ± 0.3 |
| G13S, A105N |  |  |  |  |  |  | 2.1 | 0.6 1.2 | 2.9* ± 0.4 | 1.4* ± 0.5 |
| G13S, Y139T |  |  |  |  |  |  | 1.2 | 0.2 0.2 | 0.8 ± 0.5 | 1.4* ± 0.4 |
| A105N, Y139T |  |  |  |  |  |  | 1.4 | 0.2 0.3 | 0.4 ± 0.4 | 3.6* ± 0.4 |

^1^: s: surface exposed; b: buried;

^2^: significant transcript changes compared to wtLipA above the cutoff of 2.2 and a *p*-value < 0.05;

*: significantly increased relative extracellular specific lipase activity or extracellular lipase amount compared to wtLipA with a *p*-value < 0.05.
