# Supplementary material for: Contribution of single amino acid and codon substitutions to the production and secretion of a lipase by Bacillus subtilis
Source: Microb Cell Fact. 2017 Sep 25;16:160. doi: 10.1186/s12934-017-0772-z (PMC5613506; doi:10.1186/s12934-017-0772-z)
Supplement: Supplementary file 2 — Additional file 2. Additional methods. [file 12934_2017_772_MOESM2_ESM.doc]

**Additional Methods**

#### *B. subtilis* wtLipA production analysis

*B. subtilis* TEB1030 with the plasmid pBSlipA encoding for wtLipA was cultivated as described for the 48-well FlowerPlate® cultivation in the manuscript´s method section. 1 ml cells were harvested after 2, 4, 6, 8, 10, and 24 h by centrifugation (room temperature, 21,000 g, 5 min). The culture supernatant and the cells, resuspended in 50 mM Tris-HCl pH 8, were used for a lipase activity assay as described in the manuscript´s method section. For online biomass measurement by scattered light (O.D._600nm_), replicates were prepared in 48-well Flowerplates and cultivated in the BioLector® (m2p-labs, Germany) under identical conditions (37 °C, 1,100 rpm) for 24 h.

**Protein TCA-NaDoc precipitation**

A sample volume of 1 ml was mixed with 100 µl cold 10 % (w/v) NaDoc (sodium desoxycholate) and incubated on ice for 10 min. After addition of 100 µl cold 40 % (v/v) TCA and incubation on ice for 20 min, the sample was centrifuged at 4 °C, 21,000 g for 30 min. The supernatant was discarded and the pellet containing the proteins was washed with 500 µl 80 % (v/v) acetone. After discarding the supernatant, the pellet was dried for 5 min. The pellet was resuspended in 50 mM Tris-HCl pH 8 and 2x SDS sample buffer (50 mM Tris-HCl pH 6.8, 4 % (w/v) SDS, 10 % (v/v) glycerol, 2 % (v/v) β-mercaptoethanol, 0.03 % (w/v) Bromophenol blue) to a concentration corresponding to a cell density of O.D._580nm_ = 15 and boiled for 10 min.

**Protein separation by SDS-PAGE**

Boiled samples were loaded onto a 5 % stacking gel (2.8 ml *A. dest.*, 0.83 ml 37 % (v/v) acrylamide, 1.3 ml Tris-HCl pH 6.8 (0.5 M), 50 μl 10 % (w/v) SDS, 50 μl 10 % (w/v) APS, 5 μl TEMED) on top of a 16 % separation gel (2.1 ml *A. dest.*, 5.3 ml 37 % (v/v) acrylamide, 2.5 ml Tris-HCl pH 8.8 (0.5 M), 100 μl 10 % (w/v) SDS, 100 μl 10 % (w/v) APS, 10 μl TEMED). Discontinuous SDS-gel electrophoresis was carried out at 100 V for 15 min and at 200 V for 40 min using the gadget „Mini Protean II Dual Slap Cell“ (BioRad Laboratories GmbH, Germany) and SDS running buffer (0.025 M Tris, 0.2 M glycine, 0.003 M SDS).

**Immunodetection of proteins *via* Western blotting**

Proteins from SDS gels were electrophoretically transferred at 150 mA for 15 min, and at 300 mA for 60 min onto a polyvinylidene difluoride (PVDF) membrane in a Mini-Protean 3 Cell (BioRad Laboratories GmbH, Germany) in 1 x Dunn carbonate buffer (0.003 M Na_2_CO_3_, 0.01 M NaHCO_3_) with 20 % (v/v) methanol. The PVDF membranes were washed in methanol and *A. dest.* for 1 min before protein transfer. The membrane was blocked with 3 % (w/v) bovine serum albumin dissolved in TBST (0.025 M Tris, 0.15 M NaCl, 0.0015 M KCl, 0.02 % (v/v) Tween 20) at 4 °C for 16 h. The membranes were incubated with a specific polyclonal LipA antibody (Eurogentec, Germany; produced in rabbits immunized with *B. subtilis* LipA overproduced in *E. coli* BL21(DE3)) in dilution of 1:20,000 in TBST and a second antibody goat-anti-rabbit HRP conjugate (BioRad Laboratories GmbH, Germany) in dilution 1:5,000 in TBST for 1h. After each antibody incubation step, the membranes were washed in TBST at room temperature for 30 min and 3 x 10 min. All incubation steps were accomplished on an orbital mixer. Signals were detected using freshly prepared ECL solution and the Stella 3200 Imaging System (Raytest, Germany). The ECL solution was prepared by mixing 1 ml of 4 °C cold solution A (0.025 % (w/v) luminol, 0.1 M Tris-HCl pH 8.6) with 100 µl solution B (0.1 % (w/v) *p*-hydroxy coumarate in 100 % DMSO) and 0.3 µl solution C (30 % H_2_O_2_).

**Constraint Network Analysis (CNA)**

For CNA, a protein is represented as a constraint network, where atoms are nodes and covalent and non-covalent interactions form constraints connecting the nodes [1]. The constraints in the network are modeled with different numbers of bars depending on the type and strength of the interaction. Taking into account that the network nodes are considered bodies with six degrees of freedom, covalent single bonds are modeled as five bars (leaving the rotational degree of freedom unlocked), double and peptide bonds as six bars (freezing any relative motion between two bodies), non-covalent hydrogen bonds (including salt bridges) are modeled as five bars, and hydrophobic interactions as two bars. The hydrogen bond energy (*E*_HB_) for all hydrogen bonds is computed according to a potential by Dahiyat *et al.* [2]. For thermal unfolding simulations [3, 4], hydrogen bonds are removed from the network in increasing order of their strength: A hydrogen bond is discarded from the network if *E*_HB_ > *E*_cut_. In the present study, *E*_cut_ was varied from -0.1 kcal mol^-1^ to -0.4 kcal mol^-1^ (according to 302 K to 380 K [4]) with a step size of 0.1 kcal mol^-1^ (2 K), as done previously for investigation of the thermostability of LipA [5]. For each network state generated that way, rigid and flexible regions are determined by the program FIRST [6], and from this the local index
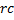
_ij_*_,neighbor_* [7, 8].
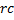
_ij_*_,neighbor_*, a neighbor stability map, characterizes the local rigidity of a protein. For improving the robustness of the analyses [9], CNA was performed on ensembles of network topologies (ENT) generated by the ENT^FNC^ approach, as done previously [5, 10]. The parameter
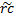
_ij_*_, neighbor_* was then computed as the median of
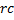
_ij_*_,neighbor_* averaged over the respective 5,000 conformations.
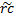
_ij_*_, neighbor_* is related to the thermodynamic thermostability of a protein [8].

1. Pfleger C, Rathi PC, Klein DL, Radestock S, Gohlke H. Constraint Network Analysis (CNA): A python software package for efficiently linking biomacromolecular structure, flexibility, (thermo-)stability, and function. J Chem Inf Model. 2013;53:1007–15. http://dx.doi.org/10.1021/ci400044m.

2. Dahiyat BI, Benjamin Gordon D, Mayo SL. Automated design of the surface positions of protein helices. Protein Sci. 1997;6:1333–7. http://dx.doi.org/10.1002/pro.5560060622.

3. Radestock S, Gohlke H. Exploiting the link between protein rigidity and thermostability for data-driven protein engineering. Eng Life Sci. 2008;8:507–22. http://dx.doi.org/10.1002/elsc.200800043.

4. Radestock S, Gohlke H. Protein rigidity and thermophilic adaptation. Proteins Struct Funct Bioinforma. 2011;79:1089–108. http://dx.doi.org/10.1002/prot.22946.

5. Rathi PC, Fulton A, Jaeger K-E, Gohlke H. Application of rigidity theory to the thermostabilization of lipase A from *Bacillus subtilis*. PLoS Comput Biol. 2016;12:e1004754. http://dx.doi.org/10.1371/journal.pcbi.1004754.

6. Jacobs DJ, Rader AJ, Kuhn LA, Thorpe MF. Protein flexibility predictions using graph theory. Proteins Struct Funct Genet. 2001;44:150–65. http://dx.doi.org/10.1002/prot.1081.

7. Pfleger C, Radestock S, Schmidt E, Gohlke H. Global and local indices for characterizing biomolecular flexibility and rigidity. J Comput Chem. 2013;34:220–33. http://dx.doi.org/10.1002/jcc.23122.

8. Rathi PC, Jaeger K, Gohlke H. Structural rigidity and protein thermostability in variants of lipase A from *Bacillus subtilis*. PLoS One. 2015;1–24. http://dx.doi.org/10.1371/journal.pone.0130289.

9. Rathi PC, Radestock S, Gohlke H. Thermostabilizing mutations preferentially occur at structural weak spots with a high mutation ratio. J Biotechnol. 2012;159:135–44. http://dx.doi.org/10.1016/j.jbiotec.2012.01.027.

10. Pfleger C, Gohlke H. Efficient and robust analysis of biomacromolecular flexibility using ensembles of network topologies based on fuzzy noncovalent constraints. Structure. 2013;1–10. http://dx.doi.org/10.1016/j.str.2013.07.012.
