# Supplementary material for: Contribution of single amino acid and codon substitutions to the production and secretion of a lipase by Bacillus subtilis
Source: Microb Cell Fact. 2017 Sep 25;16:160. doi: 10.1186/s12934-017-0772-z (PMC5613506; doi:10.1186/s12934-017-0772-z)
Supplement: Supplementary file 3 — Additional file 3. Additional figures. [file 12934_2017_772_MOESM3_ESM.doc]

**Additional Figures**

**
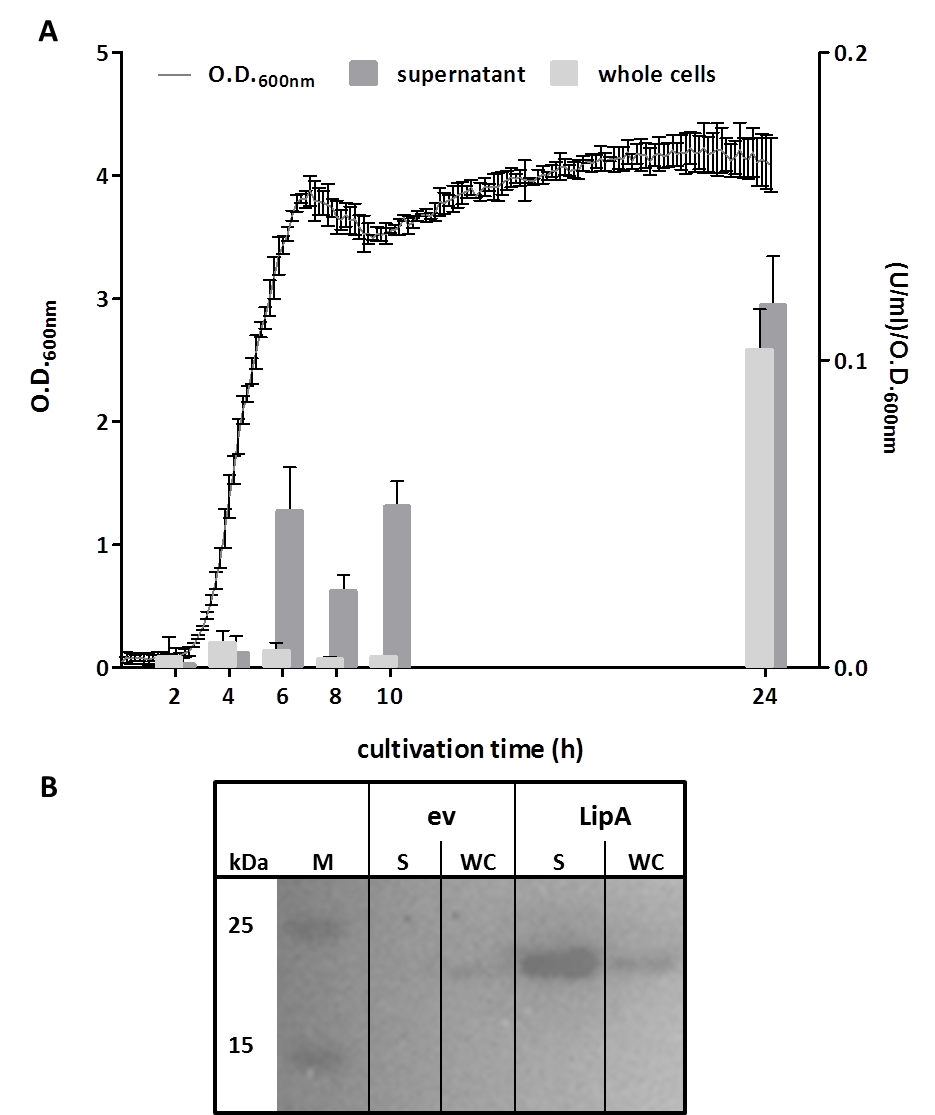
**

## Figure S1 Microfermentation of *B. subtilis* TEB1030 producing wtLipA

**A Wild-type LipA production analysis in *B. subtilis* TEB1030.** *B. subtilis* TEB1030 producing wtLipA was cultivated for 24 h in a microfermentation system using a 48 well Flowerplate® and online biomass measurement was performed in the BioLector®. The cultivation time (h) is plotted against the optical density at 600 nm on the left handed y-axis and against the volume activity normalized to the optical density ((U/ml)/O.D. _600nm_). The grey line with error bars show *B. subtilis* TEB1030 growth producing wtLipA (O.D._600nm_). After 2, 4, 6, 8, 10 and 24 h of cultivation samples were taken to determine the lipase activity in the *B. subtilis* culture supernatant (bars in dark grey) and the *B. subtilis* whole cells (bars in light grey) that was normalized to the *B. subtilis* growth at the corresponding sampling time point. **B Western Blot analysis of *B. subtilis* whole cells and culture supernatant after 6 h of wtLipA production.** The culture supernatants of *B. subtilis* TEB1030 harboring the empty vector pBSMul1 (ev) and the *lipA* expression vector pBSlipA (LipA) were precipitated with trichloroacetic acid. The precipitated culture supernatant (S) and the whole cells (WC) were resuspended in 50 mM Tris-HCl pH 8 to an O.D._580nm_ of 15. 10 µl of each sample were applied on a 16 % discontinuous SDS-PAGE together with a molecular weight standard (M). Immunodetection was performed using a specific polyclonal LipA antibody.


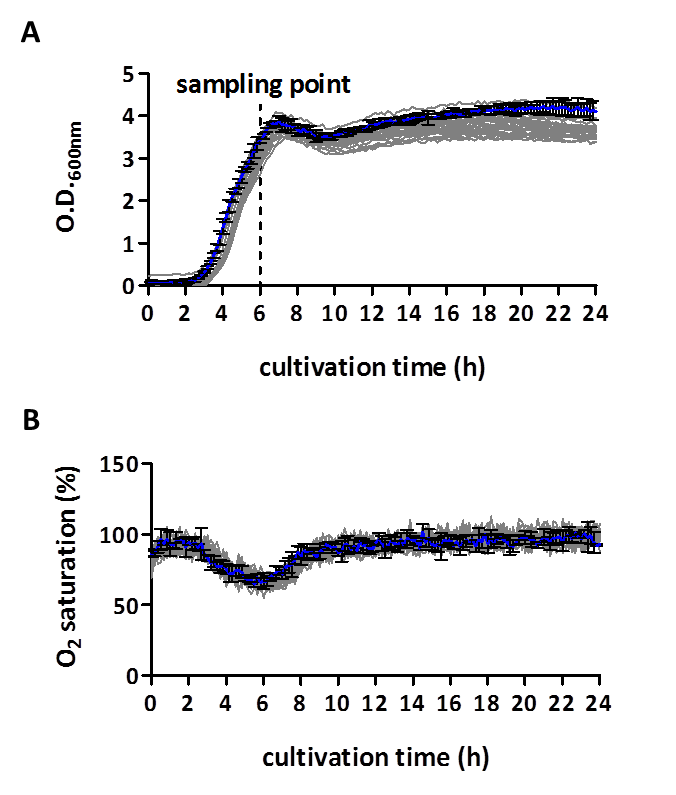


## Figure S2 Microfermentation of *B. subtilis* TEB1030 producing the 38 different LipA variants

**A 24 h online biomass measurement.** *B. subtilis* TEB1030 harboring the 38 different LipA variants were cultivated in a microfermentation system using 48 well FlowerPlates®**.** Online biomass measurement was performed for 24 h in the BioLector®. The cultivation time (h) is plotted against the optical density at 600 nm (O.D. _600nm_). The blue line indicates wtLipA optical density with corresponding error bars in black. The sampling point after 6 h of LipA production is marked. **B Oxygen saturation during microfermentation.** The cultivation time (h) of *B. subtilis* TEB1030 harboring the 38 different LipA variants is plotted against the percentaged oxygen saturation (%). The blue line indicates wtLipA oxygen saturation with corresponding error bars in black.
